# Developmental stage variation in the gut microbiome of South China tigers

**DOI:** 10.3389/fmicb.2022.962614

**Published:** 2022-11-09

**Authors:** Xianfu Zhang, Yanxin Liao, Tao Qin, Jinghua Ma, Jianxun Liu, Jianqiang Zou, Haijun Huang, Xiaojun Zhong, Menghua Yang

**Affiliations:** ^1^Key Laboratory of Applied Technology on Green-Eco-Healthy Animal Husbandry of Zhejiang Province, Zhejiang Provincial Engineering Laboratory for Animal Health Inspection and Internet Technology, College of Animal Science and Technology, College of Veterinary Medicine, Zhejiang A & F University, Hangzhou, China; ^2^State Key Laboratory of Subtropical Silviculture, College of Forestry and Biotechnology, Zhejiang A & F University, Hangzhou, China; ^3^Hangzhou Safari Park, Hangzhou, China

**Keywords:** South China tiger, Amur tigers, Bengal tigers, gut microbiome, developmental stage

## Abstract

South China tigers (*Panthera tigris amoyensis*, SC) are the most threatened tiger subspecies in the world. All the living SCs are captive in zoos or reserves and depend on artificial feeding. The composition of the gut microbiome plays an important role in sustaining the health of the host. A comprehensive understanding of the composition and development of the microbial community of SC is helpful to improve the feeding of captive SC. In this study, we collected 47 fecal samples, 37 of which were from SC of three developmental stages, 5 from adult Amur tigers (Am), and 5 from adult Bengal tigers (Bg), which were all housed in the same zoo. We investigated the diversity, richness, and composition of the bacterial microbiomes and we found that the gut microbiome of SC is strongly affected by host aging. The composition of the gut microbiome of juvenile SC experienced dramatic changes from 5 months old to 1 year old, and it showed much less difference when compared to the samples of 1 year old and the subadult. No significant differences were observed between the samples of subadult and the adult groups. The predominant phylum of 5-month-old SC is *Fusobacteriota* (33.99%) when the juvenile tigers were older than 5 months, and *Firmicutes*, but not *Fusobacteriota*, became the predominant phylum of bacteria in their gut. The gut microbiome of SC, Am, and Bg is possibly affected by their genetic variation; however, the core microbiome of these three subspecies is the same. Our data suggest that the gut microbiome of SC undergoes a developmental progression: a developmental phase (cub), a transitional phase (subadult), and a stable phase (adult). These results expand our understanding of the role of age in the development of the gut microbiome of SC.

## Introduction

The gut provides a niche for the growth and proliferation of a diversity of microorganisms. There is a growing appreciation of the influence of the gut microbiome on many functions that are important for animal health, including intestinal homeostasis, metabolism, immune maturation, physiology, and tolerance (Turnbaugh et al., 2007; Sekirov et al., 2010; Mehra et al., 2020). Disruption or dysbiosis of the gut microbiome has a significant impact on animal health and has been associated with multiple metabolic, immunological, and developmental disorders (Darmasseelane et al., 2014; Langdon et al., 2016; Marchesi et al., 2016). For example, dysbiosis of the gut microbiome caused by antibiotic treatment is associated with an increased risk of allergies, asthma, eczema, inflammatory bowel disease (IBD), and necrotizing enterocolitis (NEC) in humans (Li et al., 2014). The microbial community composition in the gut is potentially shaped by intrinsic host genetics and extrinsic environmental factors, including habitat, diet, disease, medication, and others (Ley et al., 2008; Xia et al., 2014; Nishida and Ochman, 2018). For example, infants delivered *via* cesarean section have delayed microbiome development and have an increased risk for atopic diseases, such as asthma, type 1 diabetes, coeliac disease, and food allergies, compared to those delivered vaginally (Collado et al., 2012; Li et al., 2014; Bokulich et al., 2016). Conversely, the risk for all these diseases decreases with probiotic exposure and breastfeeding (Li et al., 2014).

The tiger (*Panthera tigris*) is a large nocturnal mammal and is one of the largest Felidae species that contains six extant subspecies (Amur, northern Indochinese, Malayan, Sumatran, Bengal, and South China tigers) and three extinct subspecies (Bali, Javan, and Caspian tigers) (Rainer et al., 2004). Tigers are classified as endangered and their populations decrease every year (Luo et al., 2019). South China tigers (*Panthera tigris amoyensis*) are a unique subspecies of the tiger that occurs in China and are the most threatened tiger subspecies, probably going extinct in the wild in the 1970s. Only about 150 South China tigers are now captive in zoos or reserves (Liu et al., 2013). However, animals that are intensely managed or in captivity experience a range of changes, such as diet, living strategies, habitat, and drug treatment, which may significantly alter their gut microbiomes (Wei et al., 2019). It has been reported that captive Amur tigers have higher alpha diversity in gut microbiota, but the average unweighted UniFrac distance of bacterial taxa among wild Amur tigers was much larger (Ning et al., 2020). It was also observed in other endangered animals, such as black howler monkeys (*Alouatta pigra*), non-human primates, and sea lions (*Neophoca cinerea*), that the gut microbiome changed dramatically between captive and wild populations (Amato et al., 2013; Clayton et al., 2016; Delport et al., 2016). Therefore, it is important to have a detailed understanding of the gut microbiome of the captive South China tigers in different developmental stages, especially during the juvenile stage, because the mother tigers are often unwilling to care for their cubs when kept in zoos (Shizhou et al., 2013; Hampson and Schwitzer, 2016; Jiang et al., 2020). Tiger cubs often die of indigestion, gastrointestinal, or other diseases because they become dependent on artificial feeding (Shizhou et al., 2013; Hampson and Schwitzer, 2016; Lange et al., 2017).

In this study, we conducted analyses of the fecal microbial diversity to investigate the gut microbiota of captive South China tigers in three developmental stages, the cub (from 5 months old to 1 year old), subadult (between 2 and 3 years old), and adult (over 5 years old). We attempted to determine how the composition of the gut microbiota of captive South China tigers develops when they are aging. A detailed understanding of the gut microbiome will have important implications for the management of endangered animal health in captivity.

## Materials and methods

### Fecal sampling collection

A total of 26 healthy tigers were sampled, including 5 adult Bengal tigers (Bg), 5 adult Amur tigers (Am), and 16 Southern China tigers (SC) (3 cubs, 8 subadults, and 5 adults). The tigers were kept in the Hangzhou Wildlife Park (E: 119.9852°, N: 30.1533°). Age, sex, and species are presented in Supplementary Table 1. The tigers were divided into three groups by age: the cub group (from 5 months old to 1 year old), the subadult group (between 2 and 3 years old), and the adult group (over 5 years old). The food that the tigers consumed is described in Supplementary Table 2. Nutritional supplements were fed to tigers for the prevention and treatment of various diseases caused by vitamin and mineral deficiencies. Fresh fecal samples were collected in sterile containers and stored at −80°C for further analysis. Especially, in the cub’s group, feces were collected monthly from 5 months until 1 year old, and the groups of the samples were named M5–M12, respectively. The information on the groups is listed in Supplementary Table 1.

### Deoxyribonucleic acid extraction and 16S rRNA sequencing

Deoxyribonucleic acid (DNA) was extracted from the fecal samples using the QIAamp^®^ Fast DNA Stool Mini kit (QIAGEN Inc., Germany), which is designed for the rapid purification of high-quality genomic DNA from fresh or frozen stool samples. PCR amplification was performed using specific primers for the v3–v4 variable region of the 16S rRNA gene: 338F: ACTCCTACGGGAGGCAGCAG and 806R: GGA CTACHVGGGTWT AAT. Sequence libraries were generated using TruSeq^®^ DNA PCR-Free Sample Preparation kit (Illumina, USA) following the manufacturer’s recommendations and index codes were added. The library quality was assessed on the Qubit@ 2.0 Fluorometer (Thermo Scientific) and Agilent Bioanalyzer 2100 system. The library was sequenced in the Illumina NovaSeq platform and 250 bp paired-end reads were generated.

### Data analysis

Paired-end reads were assigned to samples based on their unique barcode and truncated by removing the barcode and primer sequence. The original Tags data (Raw Tags) were the paired-end data spliced after truncating the Barcode and primer sequences in the downstream data using the FLASH (V1.2.7)^1^ (Magoc and Salzberg, 2011). The Raw Data turns to the high-quality Tags data (Clean Data) (Bokulich et al., 2013) under specific filtering conditions according to the QIIME (V1.9.1)^2^ (Caporaso et al., 2010) quality controlled process. Then, the tags were compared with the reference database (Greengenes database)^3^ using the UCHIME algorithm (UCHIME)^4^ (Edgar et al., 2011) to detect chimera sequences, and then the chimera was removed (Haas et al., 2011).

Sequence analysis was performed using the Uparse software (Uparse v7.0.1001)^5^ (Edgar, 2013). Sequences with ≥ 97% similarity were assigned to the same OTU (operational taxonomic unit). The representative sequence for each OTU was screened for further annotation. For each representative sequence, the Silva database^6^ (Quast et al., 2013) was used based on the Mothur algorithm to annotate taxonomic information. To study the phylogenetic relationship of different OTUs and the difference of the dominant species in different samples (groups), multiple sequence alignments were conducted using the MUSCLE software (Version 3.8.31)^7^ (Edgar, 2004).

OTUs in each subgroup are not considered to be present unless found in at least 60% of the samples. The abundances of OTU were normalized using a standardized sequence number corresponding to the sample with the least sequences. Subsequent analyses of alpha diversity and beta diversity were performed based on these data. Alpha diversity was applied to analyze the complexity of the diversity of the sample of four indices, including Observed species, Shannon, Simpson, and ACE. All these indices were calculated and displayed with the amplicon package in the R software (Version 4.1.2). We conducted multiple group statistical tests of ANOVA and the Tukey HSD was used to test the statistical significance of alpha-diversity indices and to confirm the significance of these indicator species. For Beta diversity, principal coordinates analysis (PCoA) based on Bray-Curtis and UniFrac distances (Lozupone and Knight, 2005) was conducted to compare the gut bacterial community among the groups. The generalized linear model (GLM) using EdgeR (Robinson et al., 2010) in the “edgeR” R package was used to calculate the difference OTU between groups after a trimmed mean (TMM) normalization method of M-values and at a significance threshold of *P* cutoff < 0.05 and false discovery rate (FDR) < 0.05, and finally presented in the volcano plot. To analyze the differences between groups, the ANOVA or *t*-test and FDR correction were used to check for significant differences in genus levels, after which *p*-values less than 0.05 were selected for presentation. The significant differences in the present article were tested using R (version 4.2.1).^8^

The Linear Discriminant Analysis (LDA) Effect Size (LEfSe) was performed to figure out the statistical difference among biomarkers of different groups, and the PICRUSt was used to predict the function of the microbial community. The core OTUs microbiome was defined as the presence of OTUs in all samples of abundance greater than 0.1% and visualized with the “VennDiagram” and “UpsetR” R package after selected *via* the core_members approach from the “microbiome” R package.

## Results

### 16S rRNA sequencing description

The data were divided into two items, one containing South China tigers of all ages (Item 1), the other including Bengal Tiger, Amur tiger, and SC of adult (Item 2). After merging the paired-end reads, quality filtering on raw tags, and removing chimeric sequences, a total of 3,127,561 reads were recovered. The number of effective tags per individual sample ranged between 46,771 and 69,987. In Item 1, a total of 694 OTUs were identified at 97% sequence identity, with 22 phyla, 47 classes, 98 orders, 148 families, and 248 genera identified. The major phyla of *Firmicutes, Bacteroidota, Proteobacteria*, and *Actinobacteriota* contributed to 71.78% of the total microbiome abundance. In Item 2, a total of 941 OTUs were identified at 97% sequence identity and were successfully assessed at the 25 phylum levels. The major phyla of *Firmicutes, Bacteroidota*, and *Proteobacteria* contributed to 71.05% of the total microbiome abundance recorded for Item 2.

### Alpha diversity analysis

**FIGURE 1:**
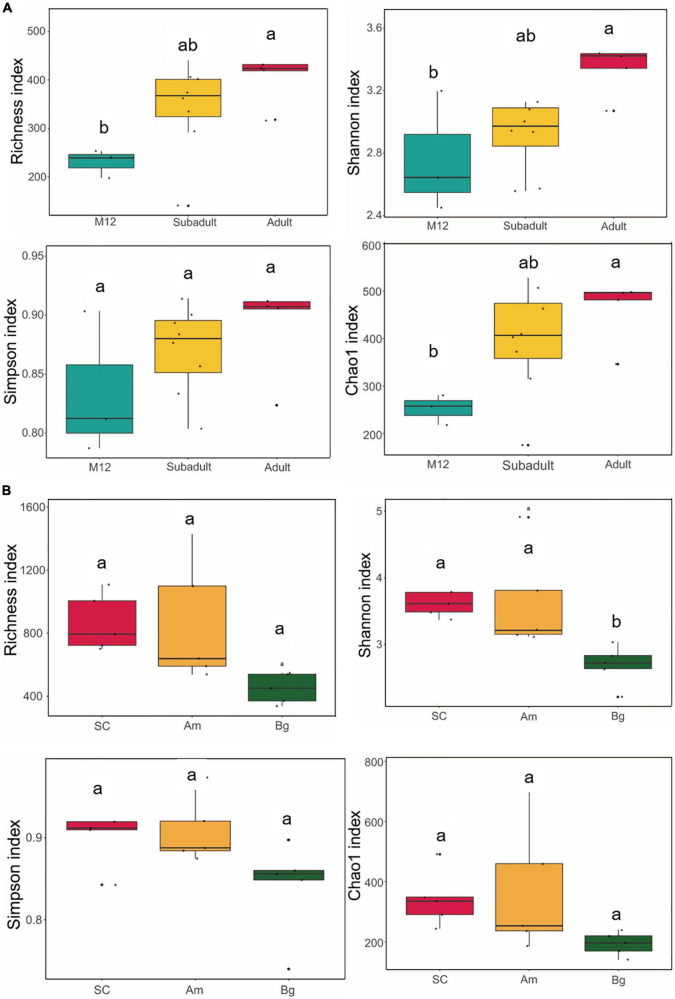
Alpha diversity of gut microbiota in South China tigers **(A)** and different subspecies tigers **(B)**. The evenness of the bacteria community was evaluated by the Shannon and Simpson indices, and the richness of the bacteria community was evaluated by the Richness and Chao1 indices. Box plots show high, low, and median values, with the lower and upper edges of each box denoting the first and third quartiles, respectively. The *x*-axis represents the information on samples. Statistical significance between different groups was indicated by a different letter (*P* < 0.05, ANOVA).

The alpha diversity is used to measure the diversity within a sample and the Shannon and Simpson indices were used to summarize the diversity in the population. To characterize the diversity of the gut microbiome of SCs, we conducted analyses of the alpha diversity of the samples of SCs in different developmental stages (cub, subadult, and adult), which included Richness, Chao1, Shannon, and Simpson indices. We found that there was no significant difference in the alpha diversity in the gut microbiome of the juvenile SCs (Supplementary Figure 1). Similar conclusions were reached for tigers of 1 year old (M12 group), subadult, and adult (Figure 1A). However, significant differences could be observed in the Richness, Chao1, and Shannon indices if compared between tigers of M12, and adult groups (Figure 1A), which indicates that as SCs are aging, the richness and evenness of their gut microbiome might gradually increase. The richness and evenness of the gut microbiome of SC are influenced by age in an accumulating way.

To find out whether the host genetic variation within species (subspecies and inheritance) has any effect on the alpha diversity of the gut microbiome of the captive SC, we compared the alpha diversities of adult SCs, Bengal tiger, Amur tiger, which were captive in the same zoo. We found that the Shannon indices of SC and Am were significantly higher than that of Bg (*p*. adj = 0.0225 and *p*. adj = 0.0324) (Figure 1B); however, no significant differences were observed in Shannon indices of SC and the Am. This result indicates that the richness and evenness of the gut microbiome of SC and Am might be higher than those of Bg.

### Beta diversity analysis

**FIGURE 2:**
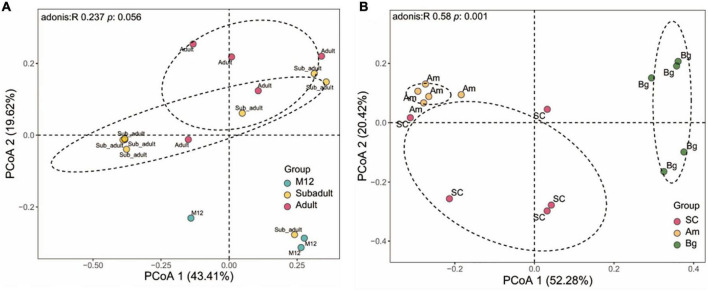
Beta diversity of gut microbiota in South China tigers **(A)** and different subspecies tigers **(B)**. Beta diversity is shown by Principal coordinate analyses (PCoA) based on Unweight UniFrac distance at the operational taxonomic unit (OTU) level. The variation explained by the plotted principal coordinates is indicated in the axis label. *P*-values of Adonis tests after adjusting by FDR are noted at the top of each PCoA plot.

The differences in species abundance distribution between samples were compared through quantitative analysis of distance in statistics. By characterizing the beta diversity of juvenile SC, we found that the samples from 6 to 8 months old (M6, M7, and M8) showed significant aggregation, and the same as the data from 10 to 12 months old (M10, M11, and M12), and they were separated from each other (Supplementary Figure 2). The samples of 5 months old (M5) were clustered together, yet, the samples of 9 months old (M9) did not show significant aggregation, and either the samples of M5 or M9 were separated from those of the rest (Supplementary Figure 2). This result indicates that within 1 year old, SC experienced different stages to shape the community of the gut microbiome. By conducting the beta diversity analysis of the data of M12, subadult, and adult, we found that the data of M12 were not clustered with either that of subadult or adult, and the data of subadult and adult groups were mixed with each other (Figure 2A). However, no significant difference of beta diversity was observed for the data from all these three groups (Figure 2A). Taking together, the results shown in Supplementary Figure 2 and Figure 2A indicate that the beta diversity of the gut microbiome of SC changes dramatically in 1 year old, and becomes stable when the tiger is older than 1 year.

We also analyzed the beta diversity of the gut microbiome of the three subspecies groups containing only adult samples, and we found that the samples from the three subspecies groups were significantly separated from one another. PCoA analysis showed that the data from SC, Am, and Bg were individually clustered (Figure 2B), which indicates that the species abundance distribution of the gut microbiome of the three subspecies tigers is different from each other, even though they are all captive in the same zoo. It strongly suggests that host genetic variation is the main factor that determines the species abundance distribution of the gut microbiome of tigers.

### The composition of the gut microbiome of South China tiger

**FIGURE 3:**
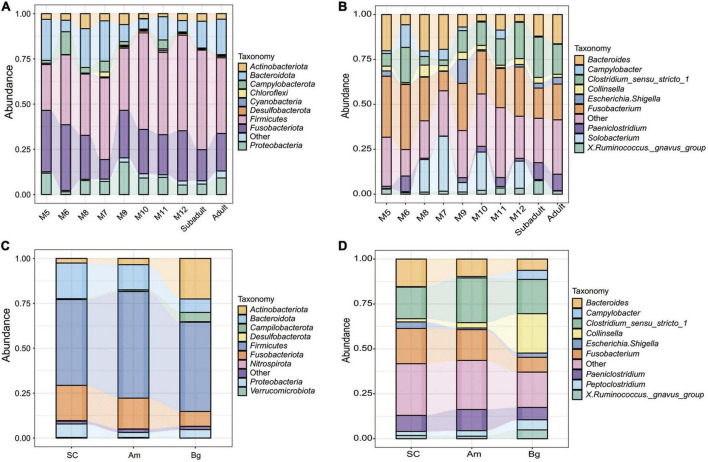
Relative abundances of gut microbiota in South China tigers at phylum **(A)** and genus **(B)** levels. Relative abundances of gut microbiota in different subspecies tigers at phylum **(C)** and genus **(D)** levels. The *x*-axis and *y*-axis represent the information of samples and relative abundance, respectively.

To determine the main composition and structure of the gut microbiome of SC, we classified the collected OTUs to the level of phylum. We found that regardless of the age or the species, the dominant phyla of bacteria in the gut microbiome of tigers were *Fusobacteriota*, *Firmicutes*, *Bacteroidota*, *Proteobacteria*, *Actinobacteriota*, and *Campylobacterota*, and *Fusobacteriota* and *Firmicutes* comprised over 60% of all the detected OTUs (Figure 3 and Supplementary Tables 3, 4). The predominant phylum of 5-month-old SC is *Fusobacteriota* (33.99%); however, when the juvenile tigers were older than 5 months, *Firmicutes*, but not, *Fusobacteriota* became the predominant phylum of bacteria in their gut (Supplementary Table 3). For all the adult tigers of different species, the predominant phylum is *Firmicutes*, which comprised over about 50% of all the detected OTUs (Figure 3C and Supplementary Table 4). Even though the dominant phyla of the gut microbiome are similar, the percentage of *Fusobacteriota* and *Bacteroidota* of SC and Am are much higher than those of Bg but the percentage of *Actinobacteriota* and *Campylobacterota* of SC and Am are much lower than those of Bg (Supplementary Table 4).

In the level of genus, we found that the most abundant genera in the gut of the tigers were *Fusobacterium, Bacteroides*, and *Clostridium_sensu_stricto_1* regardless of the age or the species, which comprised over 50% of all the detected OTUs (Figure 3 and Supplementary Tables 5, 6). For tigers of different species, the structure of the gut microbiome in the level of the genus was similar, except that the percentage of *Collinsella* and *Campylobacter* was much higher in the samples of Bengal tigers than those of the other two species (Figure 3D and Supplementary Table 6).

### Comparison of bacterial taxa among groups

**FIGURE 4:**
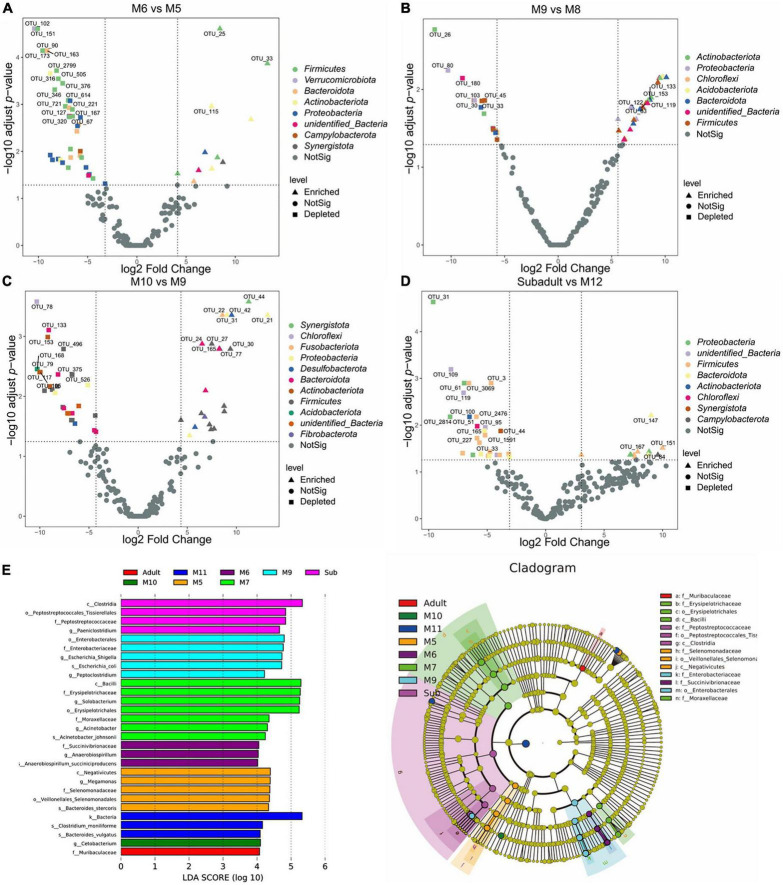
Differentially abundant microbial taxa among South China tigers of different ages. **(A)** Volcano plot illustrating the enriched and depleted OTUs between M6 and M5 groups. **(B)** Volcano plot illustrating the enriched and depleted OTUs between M9 and M8 groups. **(C)** Volcano plot illustrating the enriched and depleted OTUs between M10 and M9 groups. **(D)** Volcano plot illustrating the enriched and depleted OTUs between the subadult and M12 groups. The Volcano plot was constructed based on the abundance of OTUs (relative abundance > 1%), the symbols correspond to Enriched (square) and Depleted (triangle) OTUs, and colors represent different phyla. **(E)** The LDA using LEfSe was applied to identify biomarkers in different groups. The left histogram shows the LDA score of the biomarkers with significant differences between the groups. The circle radiating inside-out in the right cladogram indicates the classification (from phylum to genus), and the diameter is proportional to the relative abundance. The yellow circles demonstrate the species are non-significant, while others demonstrate the biomarkers by different groups.

To identify possible bacterial biomarkers associated with the difference between groups following alpha- and beta-diversity analysis, we conducted the differential abundance analysis of OTUs. Consistent with what we found in the beta-diversity analysis, significant differences in abundance were observed by analyzing the data from juvenile SC (Figure 4). Around 40–50 differentially abundant OTUs shown as volcano plots in Figure 4 were identified between the samples of M6 and M5, M8 and M9, M9 and M10, and M12 and subadult (Figure 4). Most of the differentially abundant OTUs, either depleted or enriched, were classified in the four phyla of *Firmicutes*, *Bacteroidota*, *Proteobacteria*, and *Actinobacteriota*. Much less or none of differentially abundant OTUs were observed by comparing the samples of M7 and M6, M8 and M7, M11 and M10, M11 and M12, and adult and subadult (Supplementary Figure Volcano). To further investigate differences in microbial communities among groups, we applied the LEfSe analysis with an LDA Score > 4. As shown in Figure 4E, The biomarker for the M5 group was the family *Selenomonadaceae*, for the M6 group, the family *Succinivibrionaceae*, for the M7 group, families *Erysipelotrichaceae* and *Moraxellaceae*, for M9 group, family *Enterobacteriaceae*, for the subadult group, family *Peptostreptococcaceae*, and for the adult group, family *Muribaculaceae*. These results indicated that age may have induced major shifts in the microbial community composition.

**FIGURE 5:**
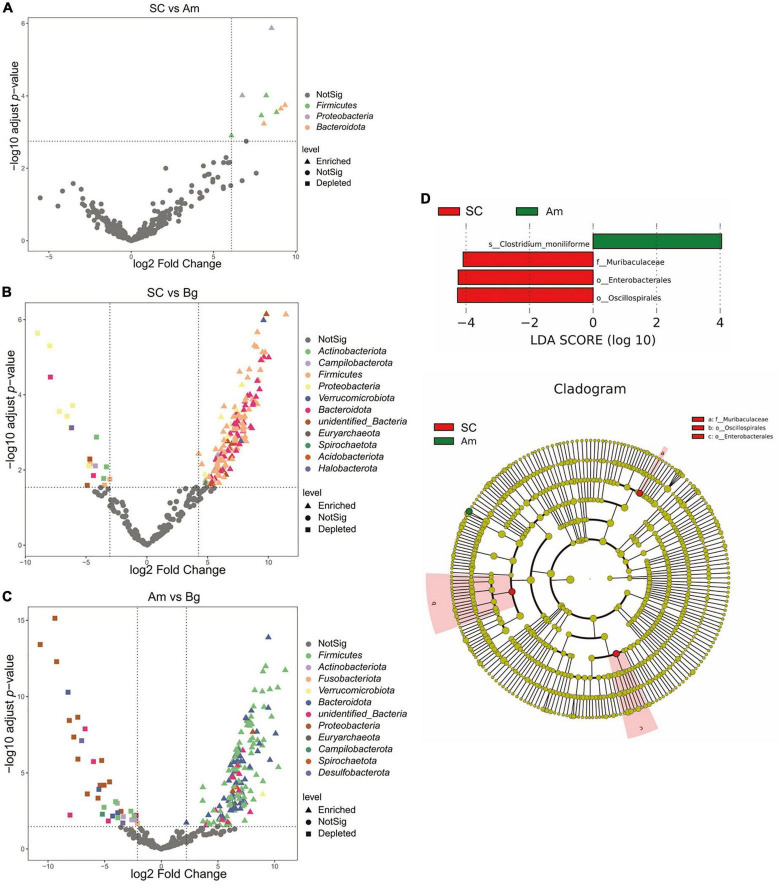
Differentially abundant microbial taxa among different subspecies tigers. **(A)** Volcano plot illustrating the enriched and depleted OTUs between SC and Am. **(B)** Volcano plot illustrating the enriched and depleted OTUs between SC and Bg. **(C)** Volcano plot illustrating the enriched and depleted OTUs between Am and Bg. The Volcano plot was constructed based on the abundance of OTUs (relative abundance > 1%); the symbols correspond to Enriched (square) and Depleted (triangle) OTUs and colors represent different phyla. **(D)** The LDA using LEfSe was applied to identify biomarkers in different groups. The left histogram shows the LDA score of the biomarkers with significant differences between the groups. The circle radiating inside-out in the right cladogram indicates the classification (from phylum to genus), and the diameter is proportional to the relative abundance. The yellow circles demonstrate the species are non-significant, while others demonstrate the biomarkers by different groups.

Differential abundance analysis of OTUs between SC and Bg found that 191 OTUs were identified, 172, which were classified as *Firmicutes* phyla, were enriched, and 19, which were classified as *Actinobacteria*, *Campilobacterota*, and *Proteobacteria* phyla, were depleted (Figure 5). A total of 196 differentially abundant OTUs were identified by comparing the group of Amur tigers and Bengal tigers, 37 of which were classified mainly as *Firmicutes* and *Bacteroidetes* phyla were enriched, and 159 of which were classified as *Firmicutes* and *Proteobacteria* phyla were depleted (Figure 5). LEfSe analysis was further applied to investigate differences in community composition among the SC, Bg, and Am groups. The results showed that the biomarkers for the SC group were of the orders *Oscillospirales* and *Enterobacterales*, as well as the family *Muribaculaceae*, while the biomarker for Am group was the species *Clostridium_moniliforme* (Figure 5D).

**FIGURE 6:**
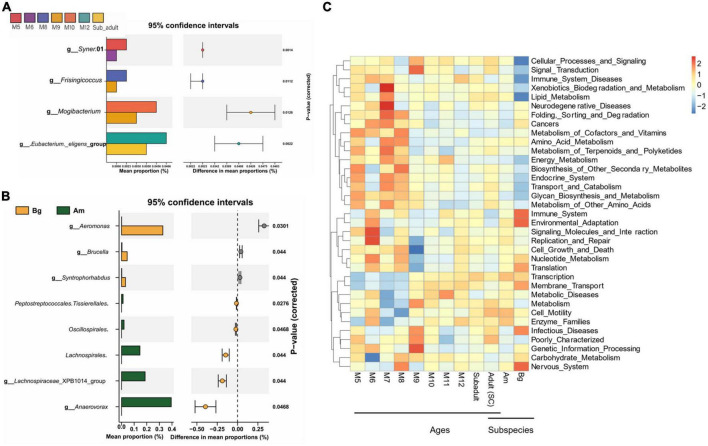
The difference in the genus and the function of the intestinal bacterial microbiome. **(A)** The difference in the genus of the intestinal bacterial microbiome among South China tigers of different ages. **(B)** The difference in the genus of the intestinal bacterial microbiome among different subspecies tigers. **(C)** The cluster heatmap shows the relative abundance of bacterial functional categories related to KEGG pathways at level 2. The bacterial functional categories were analyzed by PICRUSt based on qualified sequences.

Differential abundance analysis of OTUs at the genus level among the groups by *t*-test and adjusted by FDR are shown in Figure 6. The abundance of *Syner.*01 was significantly enriched only in the group of M5 but not in the SC samples older than 5 months (*P* < 0.05). Analysis between the groups M8 and M9, M9 and M10, and M12 and subadult showed that *Frisingicoccus* was significantly increased in the M8 group (*P* < 0.05), while *Mogibacterium* was enriched in the M10 group (*P* < 0.05) and the *Eubacterium_eligens*_group was significantly enriched in the group of M12 (*P* < 0.05). By conducting the differential abundance analysis of OTUs between groups of tigers in different subspecies, we found that at the family level, *Aeromonadaceae*, *Syntrophorhabdaceae*, and *Anaerovoracaceae* were enriched in the samples of Bg compared to that of either SC or Am (*P* < 0.05). At the genus level, *Aeromonas*, *Syntrophorhabdus*, and *Brucella* were enriched in the samples of Bg (*P* < 0.05), while *Lachnospiraceae*_XPB1014_group and *Anaerovorax* were enriched in that of Am (*P* < 0.05). At the genus level, no significant difference was observed when comparing the samples of SC with those of Bg or Am.

PICRUSt was applied to explore the variation of bacterial functional categories of the microbial communities. As shown in Figure 6C, The top 35 sub-functions of the KEGG level2 were presented by cluster heatmap. We found that the relative abundances of several sub-functions decreased with aging, such as folding, sorting and degradation, cancers, metabolism of cofactors and vitamins, and amino acid metabolism, while transcription and membrane transport sub-functions increased with aging. In addition, PICRUSt analysis showed that the relative abundance of the top 35 sub-functions from SC and Am groups was similar, whereas the Bg group had a different abundance of cellular processes and signaling, immune system diseases, lipid metabolism, immune system, environment adaption, and nervous system (Figure 6C). These results suggested that age and subspecies influence the intestinal microbial functions.

### Core microbiome

**FIGURE 7:**
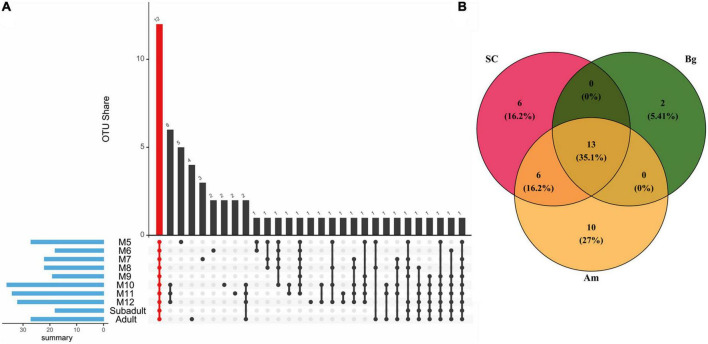
The core OTUs among different groups. **(A)** The core microbiome is shared by South China tigers of different ages. The red column indicates all shared OTUs by all ages of South China tigers. The blue column indicates the sum of core OTUs for each age group. **(B)** Venn diagram describing the numbers of OUTs among different subspecies of tigers. The intersection of the circles indicates OTUs common to different subspecies of tigers.

As the core microbiome is essential for the gut microbiome to perform its functions, we first explored the core microbiome of SC of different ages. A total of 57 OTUs occupying 90.6% of the total abundance of the gut microbiota were identified as the core microbiome of SC, among which 12 OTUs consisting were common to all groups and represented 57.1% of the total abundance (Figure 7A). The 12 common OTUs mainly included *Firmicutes* (5 OTUs), *Fusobacteriota* (2 OTUs), and *Proteobacteria* (2 OTUs) at the phylum level. At the genus level, the percentage of OTU_2 (*Fusobacterium*) was almost identical in all groups except M7. Besides, we found that the percentage of OTU_1 (*Clostridium_sensu_stricto_1*) increased, and the relative abundance of OTU_7 (*Fusobacterium*) and OTU_8 (*Bacteroides*) decreased as SC aged (Supplementary Table 7 and Supplementary Figure 3).

For different subspecies of adult tigers, 37 OTUs occupying 84.4% of the gut microbiota are identified as the core microbiome of SC, Am, and Bg (Figure 7B). A total of 13 OTUs were found in all three subspecies, which accounted for 61.0% of the total abundance. The 13 common OTUs mainly included *Firmicutes* (7 OTUs) and *Fusobacteriota* (3 OTUs) at the phylum level (Supplementary Table 8). At the genus level, the percentage of OTU _ 2 (*Fusobacterium*) was significantly enriched in SC and Am, which were about four times more abundant than that of Bg, and the relative abundance of OTU_7 (*Ruminococus_ganvus_group*) and OTU_13 (*Collonsella*) was significantly enriched in the group of Bg but not in the other two groups (Supplementary Table 8 and Supplementary Figure 4).

## Discussion

Gut microorganisms have unique metabolic functions and help the host perform both physiological and biochemical functions (Sender et al., 2016). Fecal samples are a non-invasive and sustainable method for observing gut microbiota in endangered animals. Although fecal samples cannot reflect the current dynamic changes of bacteria in the animal gut, they can still reflect the composition of the entire gut microbiome (Best et al., 2017; Li et al., 2017) which can help us to understand the structure and differences of the intestinal flora of animals. In this study, the intestinal flora of 26 healthy tigers, including 5 adult Bengal tigers, 5 adult Amur tigers, and 16 SC (3 cubs, 8 subadults, and 5 adults), was characterized by analyzing 16s rRNA amplicons from their fecal samples to determine how age and genetic variation affect the gut microbiome of SC.

### Southern China tiger gut microbiome is dominated by bacteria associated with meat digestion

As reported in other carnivorous animals (Jiang et al., 2020), the gut microbiome of SC was mainly occupied by the phyla *Fusobacteria*, *Firmicutes*, *Bacteroidota*, *Proteobacteria*, and *Actinobacteriota*. *Fusobacteria* that occupied in high percentage in the gut microbiome of SC was contributed mainly by the *Fusobacterium*, which was found to have a higher proportion of other predators’ gut microbiome, such as seals, alligators, and cheetah (Keenan et al., 2013; Nelson et al., 2013; Nishida and Ochman, 2018), and was presumed to be related to the digestion of meat (Jiang et al., 2020). *Firmicutes*, another predominate phylum of the gut microbiome of SC, which is also a typical abundant bacterial phylum in other mammals and plays a key role in the breakdown of the fiber cellulose and nutrient absorption (Xue et al., 2015), was enriched as a function of SC aging. The majority of *Firmicutes* are contributed by *Clostridium_sensu_stricto_1* at the genus level, the percentage of which also increased with the aging of SC (Supplementary Table 5). *Clostridium_sensu_stricto_1* had been reported to be associated with protein digestion in felines (Lubbs et al., 2009) and was also found in the gut of leopards (Han et al., 2019). *Bacteroides*, which is the major genus of *Bacteroidota* phylum, was also found to play an important role in protein degradation (Arumugam et al., 2011), and it was found that the ratio of *Firmicutes* and *Bacteroides* in the feces increased with aging (Haro et al., 2016), which may be associated with switching to the high-fat diet (Hildebrandt et al., 2009; Tremaroli and Backhed, 2012). In this study, we also found that the ratio of *Firmicutes* and *Bacteroides* increased from cub to adult (Supplementary Tables 3, 5). So, the three major phyla (*Fusobacteria*, *Firmicutes*, and *Bacteroidetes*) or genera (*Fusobacterium, Clostridium_sensu_stricto_1*, and *Bacteroides*) in the gut microbiome of SC were all associated with meat digestion. The gut microbiome of SC was also occupied by *Proteobacteria*, which are facultative anaerobes and promote colonization of obligate anaerobes in the intestinal niche (Shin et al., 2015) and plays a key role in the degradation of lignin and the breakdown of complex compounds in forage (Evans et al., 2011). *Proteobacteria* was also detected in other feline gut microbiota (Fang et al., 2012; Han et al., 2019; Jiang et al., 2020; Sun et al., 2022). *Actinobacteria* also contributed to the gut microbiota of the tigers, which might play a role in maintaining host homeostasis, inhibition of Gram-negative pathogens, and lactic acid fermentation (Colston and Jackson, 2016). The gut microbiome of SC within 2 months old (Sun et al., 2022) was found to occupy the similar predominant phyla as we found in this study. However, the genus that was reported in the gut of those tigers was much different from what we found here, except for the genus of *Collinsella* and *Campylobacter*, which were found in both samples (Supplementary Table 5; Sun et al., 2022). This might indicate that the structure of the gut microbiome of SC changed more dramatically in the early period after birth, and after 1 year old, it became more stable.

These major phyla, *Fusobacteria*, *Firmicutes*, *Bacteroidetes*, *Proteobacteria*, and *Actinobacteriota*, which occupied the gut microbiome of SC, were also found in the other two subspecies of tigers, Amur tigers, and Bengal tigers, which are captive in the same zoo as SC is (Supplementary Tables 3, 4). The same phyla were also reported to occupy the gut microbiome of captive Amur tigers in the northeast of China (He et al., 2018; Ning et al., 2020) and Bengal tigers living in Nepal (Karmacharya et al., 2019). Both this study and a study of the gut microbiome of tigers captive in a zoo in Southern China conducted by Jiang et al. found that *Firmicutes* was the major phylum of these three subspecies of tigers (Jiang et al., 2020). However, the major phylum of Amur tigers in the northeast of China (He et al., 2018; Ning et al., 2020) and Bengal tigers living in two specific areas of Nepal (Karmacharya et al., 2019) was *Proteobacteria*, which comprised less than 10% of the total gut microbial communities of the samples in this study. These data might indicate that the gut microbiome of tigers is mainly determined by the species; however, the environmental factors would affect the composition of the gut microbiome to some degree. Like Bengal tigers that live in different districts of Nepal, their gut microbiota profile has significant differences in the composition of microbial communities based on their geographic locations (Karmacharya et al., 2019).

The genus classified in the core microbiome is common in SC, which included samples from three developmental stages, Am and Bg, which only included the adult samples (Figure 7 and Supplementary Tables 7, 8). This indicates that the age and genetic variation of the tiger would not affect the composition of the core microbiome in the gut. The genus of *Fusobacterium* in the gut microbiome is often associated with meat digestion as reported in other predators (Ley et al., 2008; Keenan et al., 2013; Nelson et al., 2013; Nishida and Ochman, 2018). *Ruminococcus gnavus*, an early colonizer of the human gut (Kelly, 2016; Wu et al., 2021), was reported to be associated with mucin glycan utilization, which is important for the colonization of bacteria in the gut (Tailford et al., 2015; Lim et al., 2017). Species of *Bacteroides* are important for metabolizing polysaccharides and oligosaccharides, which could provide nutrition and vitamins to the host and other intestinal microbial residents (Wexler, 2007; Zafar and Saier, 2021). *Blautia* is a genus of anaerobic bacteria that shows a series of potential probiotic properties and occur widely in the feces and intestines of mammals (Liu et al., 2021). *Collinsella* is an important genus that produces ursodeoxycholate, which was previously reported to prevent COVID-19 infection (Hirayama et al., 2021). Two OTUs belonging to the gena of *Escherichia-Shigella* and *Sutterella* are specifically found only in the core microbiome of SC (Supplementary Table 6). The genus of *Sutterella* is gut-health-related beneficial taxa (Nguyen et al., 2019). Overall, the composition of the core microbiome of SC is associated with meat digestion and is important to maintain the intestinal homeostasis of the SC gut microbiome.

### The composition of South China tiger gut microbiome undergoes significant changes in early age

The richness and evenness of the gut microbiome of SC increased gradually as SC was aging as shown by the alpha diversity analysis (Supplementary Figure 1 and Figure 1). The alpha diversity showed no significant difference between each developmental stage. However, significant differences in alpha diversity were observed when the data of the M12 group were compared with that of the adult group, which indicated that the age of SC has an accumulating effect on the alpha diversity of the gut microbiome. Unlike the alpha diversity, the beta diversity of the samples of SC in 1 year old showed a significant difference (Supplementary Figure 2). By analyzing the beta diversity of the gut microbiome of the juvenile SC, we found that the data of M5 were significantly separated from those of other groups, and it is the same case as for the data of M9 (Supplementary Figure 2). For the other groups, M6, M7, and M8 were aggregated with each other and it is the same as for the data of M10, M11, and M12 (Supplementary Figure 2). The data of M12 were separated from those of subadult and adult groups, which were somehow mixed with each other (Figure 2A). This result indicated that the gut microbiome profile has significant differences in the composition of microbial communities based on the ages of SC. Comparison of bacterial taxa among groups of SC could also support what we found in the beta diversity analysis. Significant different OTUs were observed by comparing the data of M5 and M6, M8 and M9, M9 and M10, and M12 and subadult as well (Figure 4). However, no different OTUs were found among M6, M7, and M8, and is the same case as for M10, M11, and M12 (Supplementary Figure Volcano). Both the beta diversity analysis and the significantly altered species analysis suggested that the gut microbiome of the juvenile SC develops much more quickly than that of the subadult or adult SC. A study of humans from infancy to childhood found that the developing gut microbiome undergoes three distinct phases of microbiome progression (Stewart et al., 2018). Another study of juvenile SC (within 2 months old) found that the diversity, abundance, and composition of the gut microbiome of SC undergoes significant changes within 50 days after birth (Sun et al., 2022). Therefore, the data of this study might suggest that like the case in humans (Stewart et al., 2018), the gut microbiome of SC might undergo similar developmental progression: a developmental phase (cub), a transitional phase (subadult), and a stable phase (adult).

Differential comparison in the genus level found that *Syner_01* was specifically enriched in the M5 group (Figure 6). *Syner_01* was found to be the predominant genus during sulfur-driven autotrophic denitrification for the treatment of low-C/N-ratio and sulfur-laden wastewaters (Wang et al., 2021). *Frisingicoccus*, which was reported to be enriched in the gut of pigs by supplementing zinc in the diet (Rattigan et al., 2020), was specifically enriched in M6, M7, and M8 groups compared to the M9. *Mogibacterium*, which might help to attenuate ileal inflammation and would be enriched in the human gut with vitamin D3 ingestion (Valle et al., 2021), was specifically enriched in M10, M11 and M12 groups compare to M9. All the samples of the juvenile SC in this study were collected each month from three juvenile SC that lived in the same cage from 5 months old to 12 months old and they were fed the same diet, therefore the different genera found in the samples collected from different months were probably caused by the age of the juvenile SC. No different genus was found by comparing the data either of M12 and subadult or subadult and adult, however, *Eubacterium.eligens*_group was found to be enriched in M12 compared with that of adult (Figure 6), which indicated that subadult might be a transitional phase of the gut microbiome developing between cub and adult.

### Implications for Southern China tiger conservation

The gut microbiome plays a very important role in host health. Understanding the composition and function of the gut microbiome of endangered animals in different developmental stages could provide insights into possible measures to promote the health of these animals by targeting these microbial communities in clinical treatment and health management settings. Either the external factors, such as habitat, diet, disease, and medication, or the internal factors, such as age and genetic variation, would affect the composition of the gut microbiome to some extent. The age-associated changes, such as diet, habitat, behavior, and illness, would cause changes in gut microbiota. Studies in model organisms like fruit fly (Drosophila melanogaster), nematode (*Caenorhabditis elegans*), and mouse demonstrate that elderly and younger populations do show differences in gut microbe composition and the changes of the gut microbiome are indeed a causative factor of aging (Rera et al., 2012; Thevaranjan et al., 2017; Maynard and Weinkove, 2018). Jiang et al. (2020) found that the beta diversity of gut bacterial microbiota was significantly different depending on diet and age. In this study, we collected the fecal samples of three juvenile SC that lived together and were fed the same food since they were 5 months old–1 year old, and found that the composition of the gut microbiome of these three juvenile SC showed significant difference during this period (Supplementary Figure 2 and Figure 4). However, the composition of the gut microbiome from the samples of SC, which were older than 1 year (M12, subadult, and adult groups), did not show a significant difference between each other (Figure 2A and Supplementary Figure Volcano). What we found in this study strongly suggested that for the captive SC the composition of the gut microbiome of SC develops dramatically in 1 year old. However, after 1 year old, it goes into a transitional phase as in subadult and then becomes stable as in the adult. These results expand our understanding of the role of age in the development of the gut microbiome of SC and might tell us that we should pay more attention to the health of the gut microbiome of juvenile SC.

## Conclusion

In this study, we investigated the characteristics of the gut microbiome of captive SC at different ages. We found that the gut microbiome of SC, which is frequently dominated by bacteria associated with meat digestion and the composition of the dominant phyla or genus, would change with age. The data of this study demonstrate that the dramatic changes in the gut microbiome happened in juvenile SC and the gut microbiome of SC might undergo developmental progression: a developmental phase (cub), a transitional phase (subadult), and a stable phase (adult). These findings were of great significance for the feeding of captive SC, especially during the early age of the tigers. We also analyzed the composition of the gut microbiome from SC, Am, and Bg, which are all captivated in the same zoo. We found that the composition of the gut microbiome of the three subspecies tigers is different from each other, which strongly suggests that the host genetic variation is the main factor that determines the composition of the gut microbiome of tigers. In summary, this study provides comprehensive information on the gut microbiome of tigers and further describes the different effects of tiger subspecies and age on the gut bacterial community.

## Data availability statement

The datasets generated for this study can be found in the raw sequences of 16S rRNA gene that were available in the NCBI Sequence Read Archive under BioProject PRJNA838725 (Item 1) and the BioProject PRJNA838743 (Item 2).

## Author contributions

XnZ and YL isolated DNA and uploaded the data. XoZ, YL, and TQ analyzed the data. JM, JL, JZ, and HH collected the sample. MY designed the study and wrote the manuscript. All authors contributed to the article and approved the submitted version.

## Footnotes

1
http://ccb.jhu.edu/software/FLASH/

2
http://qiime.org/scripts/split_libraries_fastq.html

3
http://greengenes.lbl.gov

4
http://www.drive5.com/usearch/manual/uchime_algo.html

5
http://www.drive5.com/uparse/

6
http://www.arb-silva.de/

7
http://www.drive5.com/muscle/

8
https://www.r-project.org/

## Supplementary material

Supplementary Figure 1 Volcano grams of other subgroups of intestinal bacteria of the South China tiger.
