# Supplementary figures and images for: Developmental stage variation in the gut microbiome of South China tigers
Source: Front Microbiol. 2022 Nov 9;13:962614. doi: 10.3389/fmicb.2022.962614 (PMC9682017; doi:10.3389/fmicb.2022.962614)

# adult vs M12

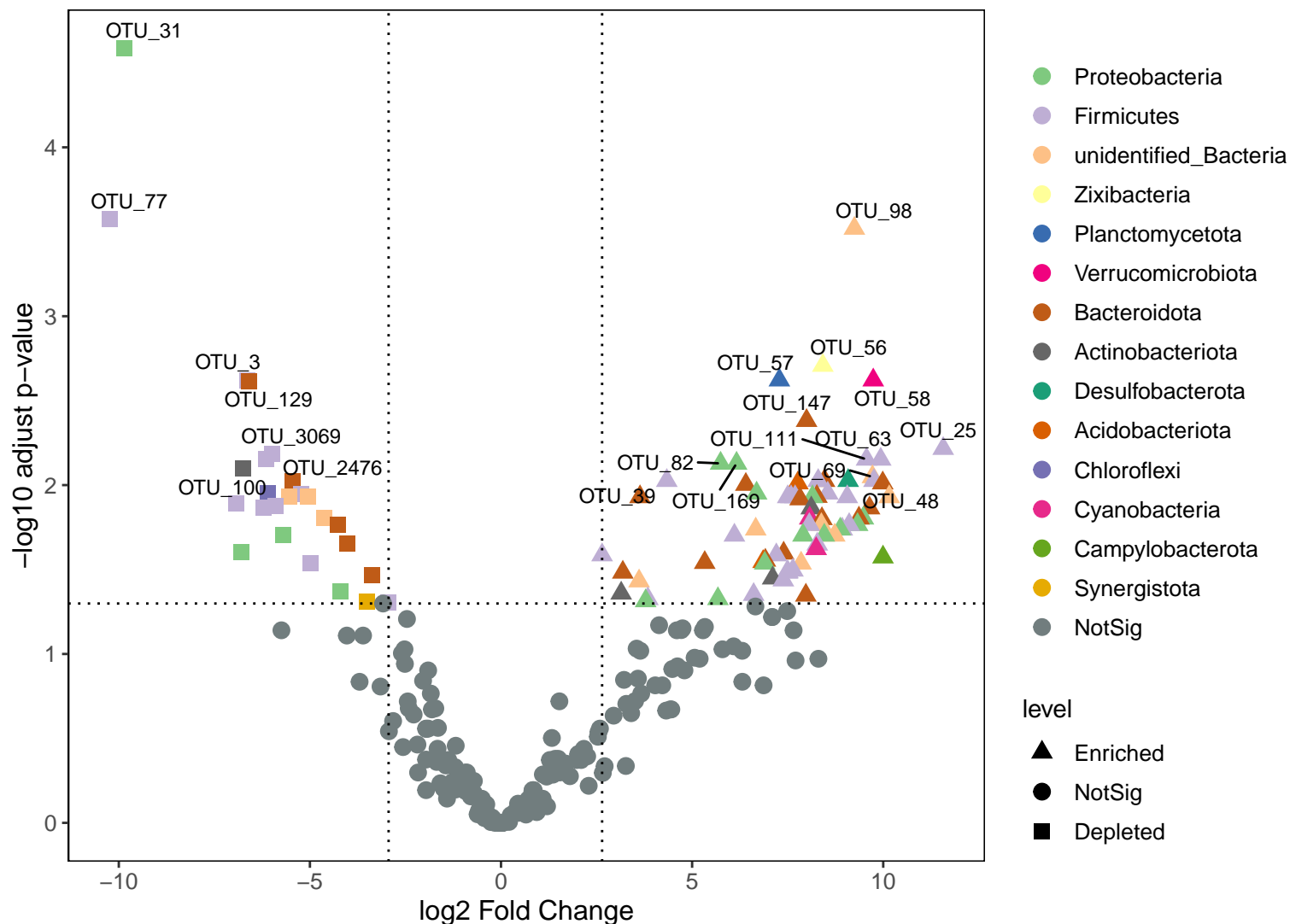

Supplement: Supplementary file 1 [file Data_Sheet_1.ZIP › Supplement Figure Volcano/adult vs M12 _Volcano plot.pdf]

M10 vs M5

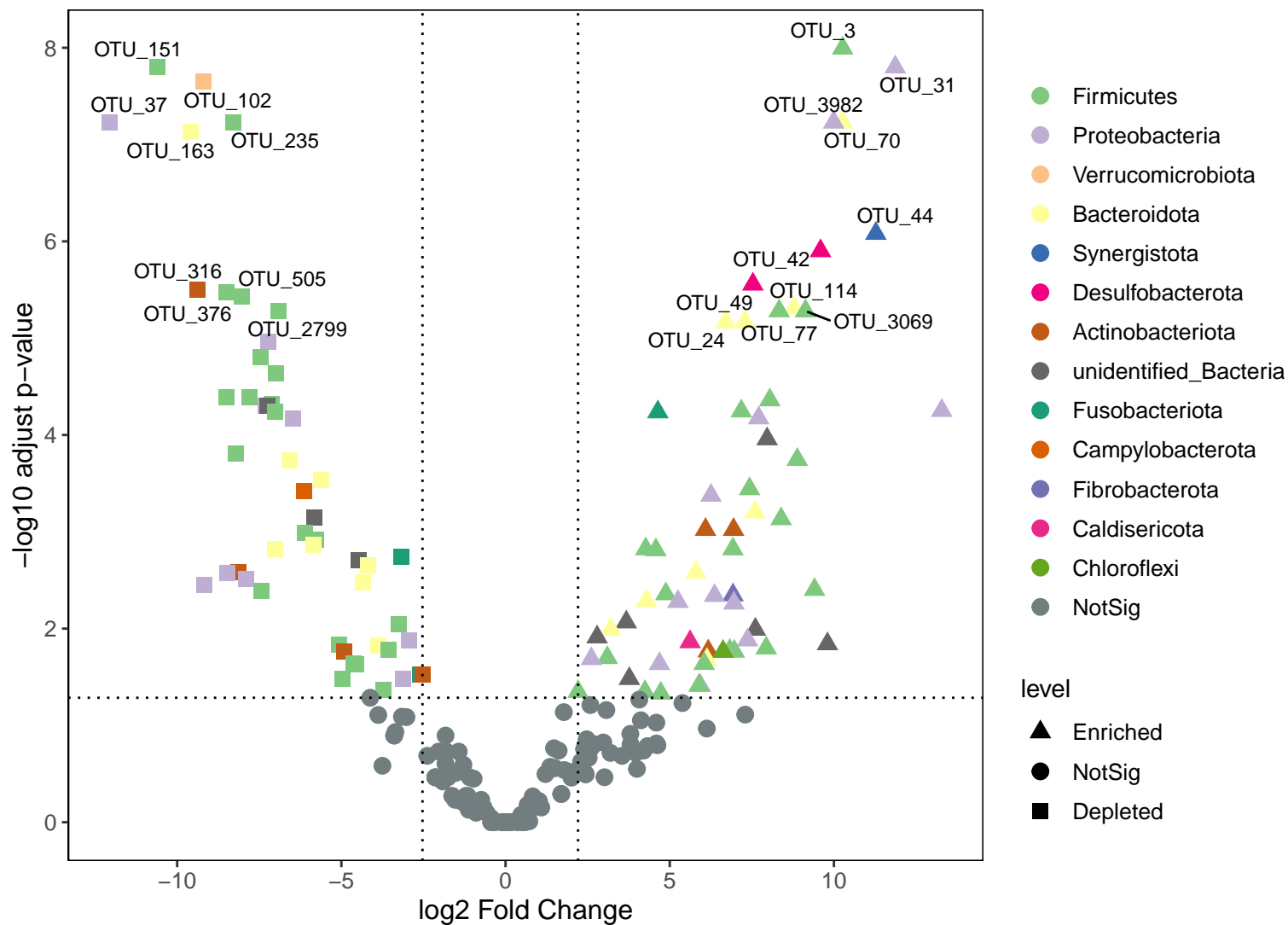

Supplement: Supplementary file 1 [file Data_Sheet_1.ZIP › Supplement Figure Volcano/M10 vs M5_Volcano plot.pdf]

M10 vs M6

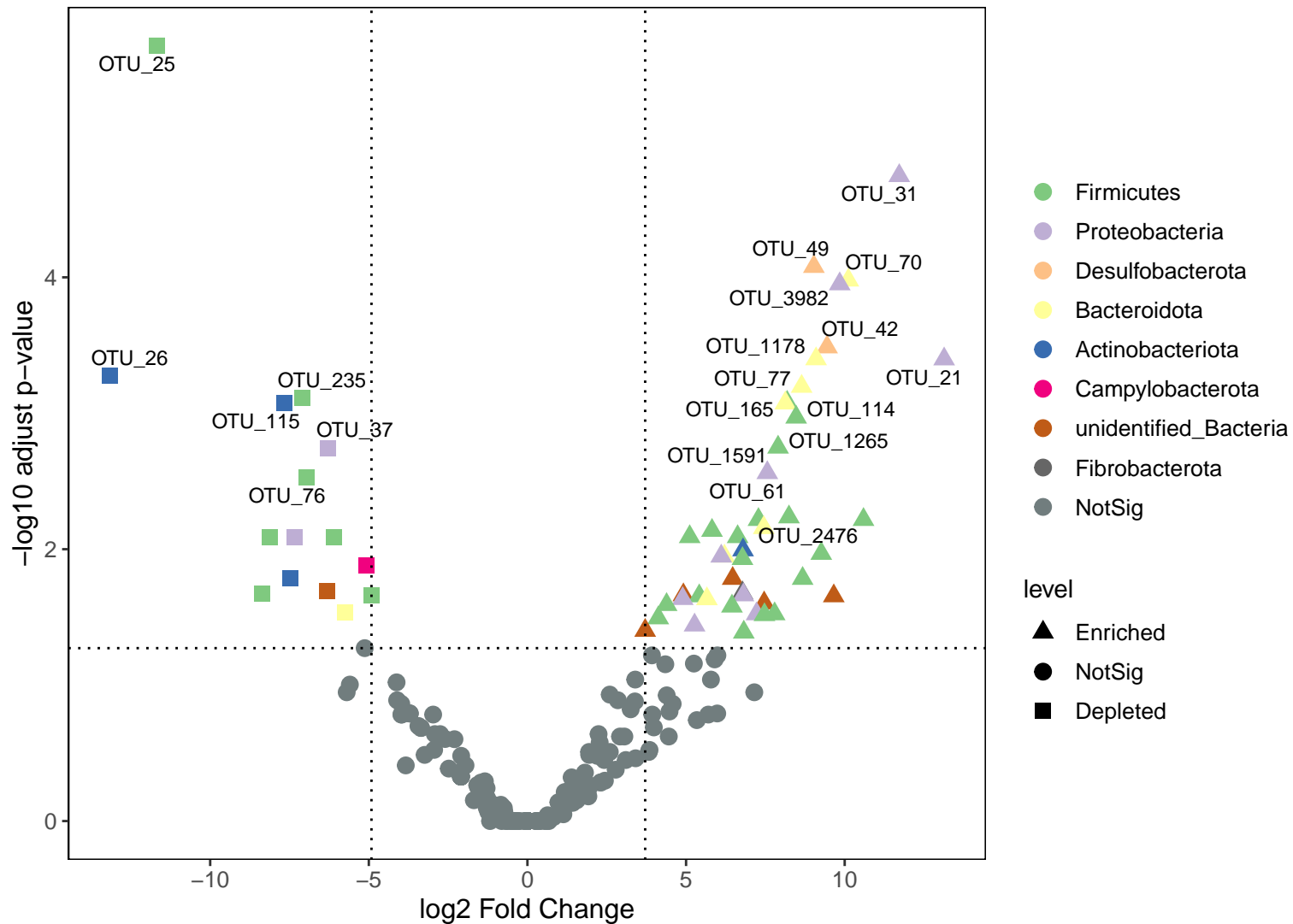

Supplement: Supplementary file 1 [file Data_Sheet_1.ZIP › Supplement Figure Volcano/M10 vs M6 _Volcano plot.pdf]

# M10 vs M7

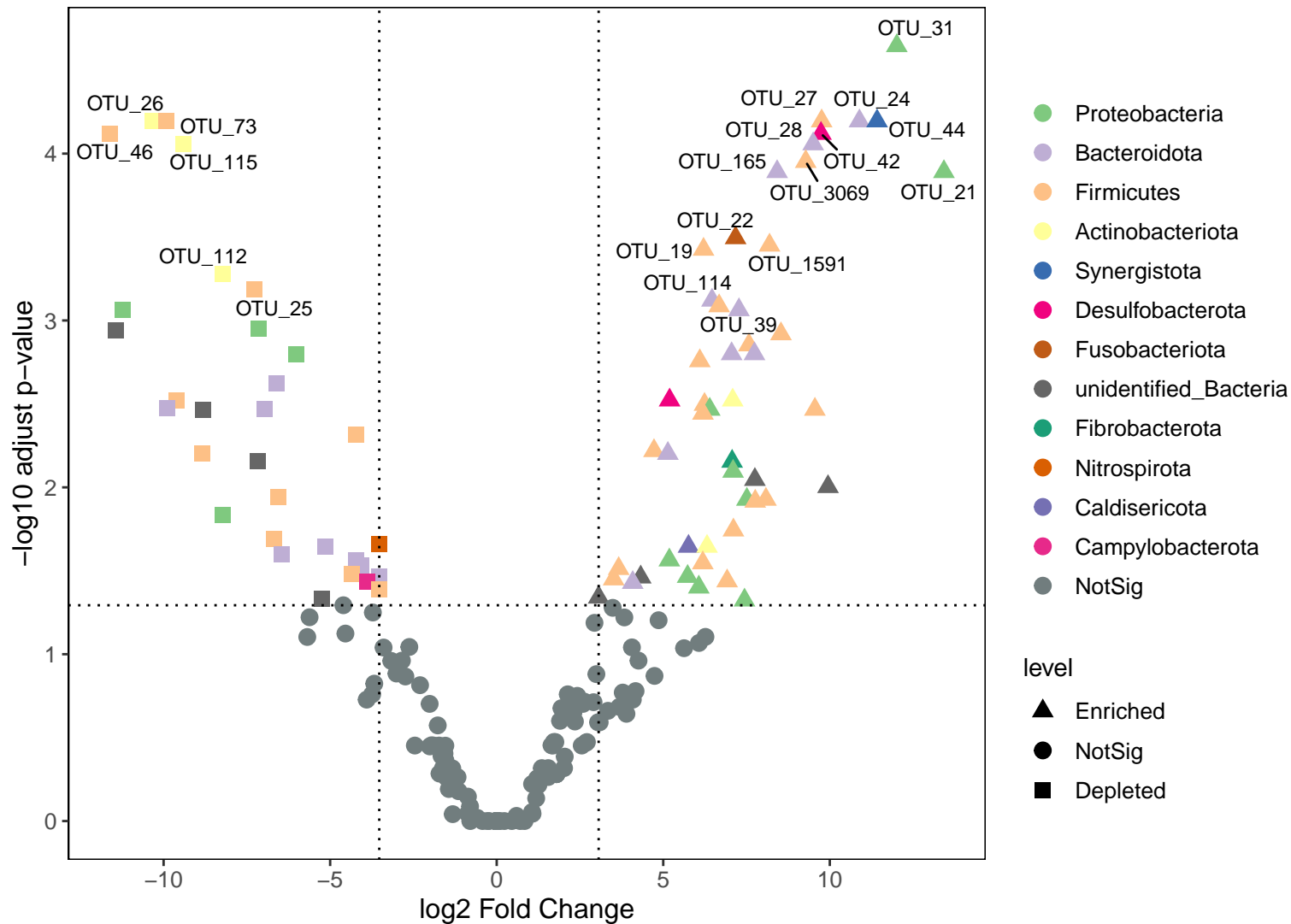

Supplement: Supplementary file 1 [file Data_Sheet_1.ZIP › Supplement Figure Volcano/M10 vs M7 _Volcano plot.pdf]

M10 vs M8

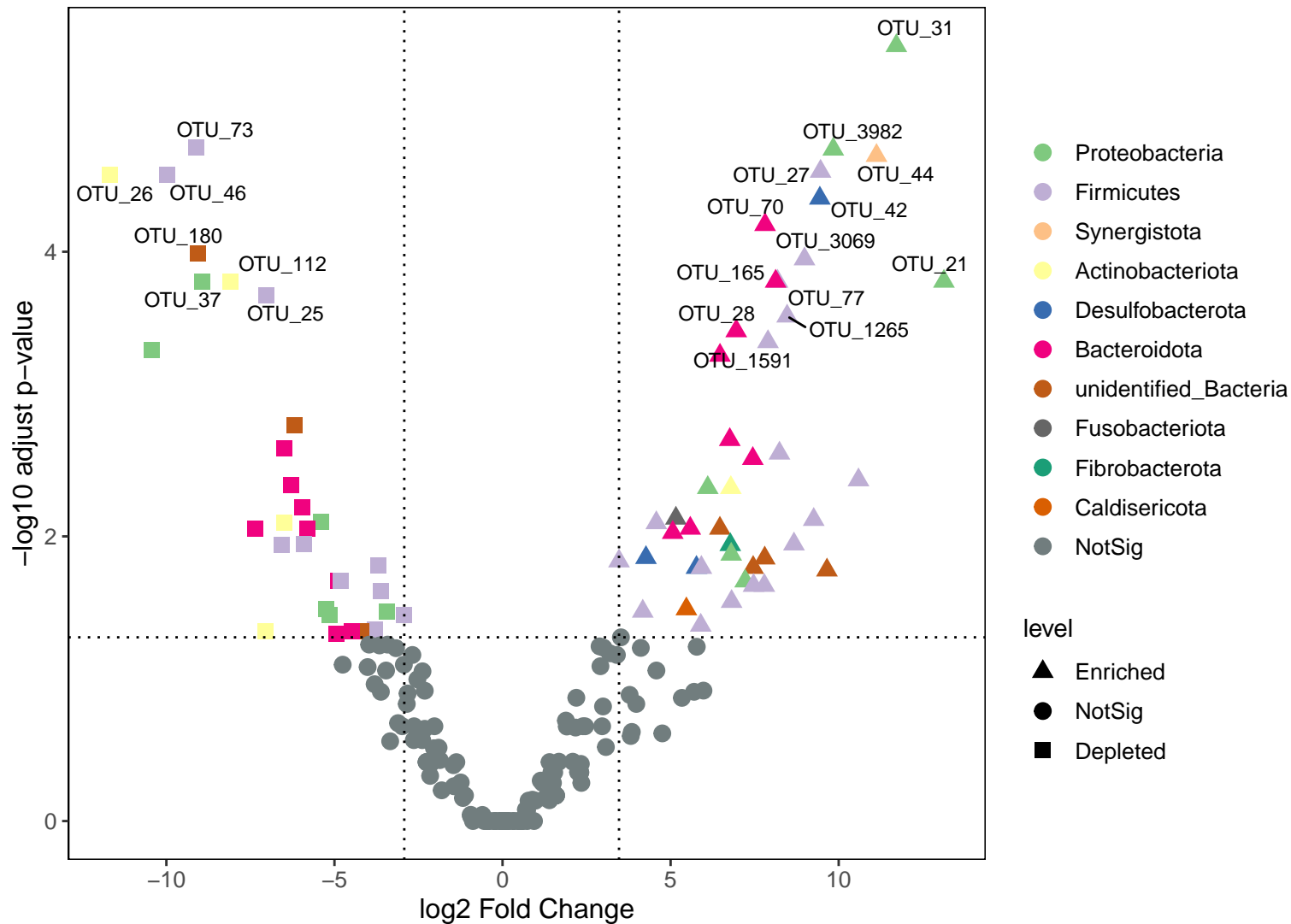

Supplement: Supplementary file 1 [file Data_Sheet_1.ZIP › Supplement Figure Volcano/M10 vs M8 _Volcano plot.pdf]

M10 vs M9

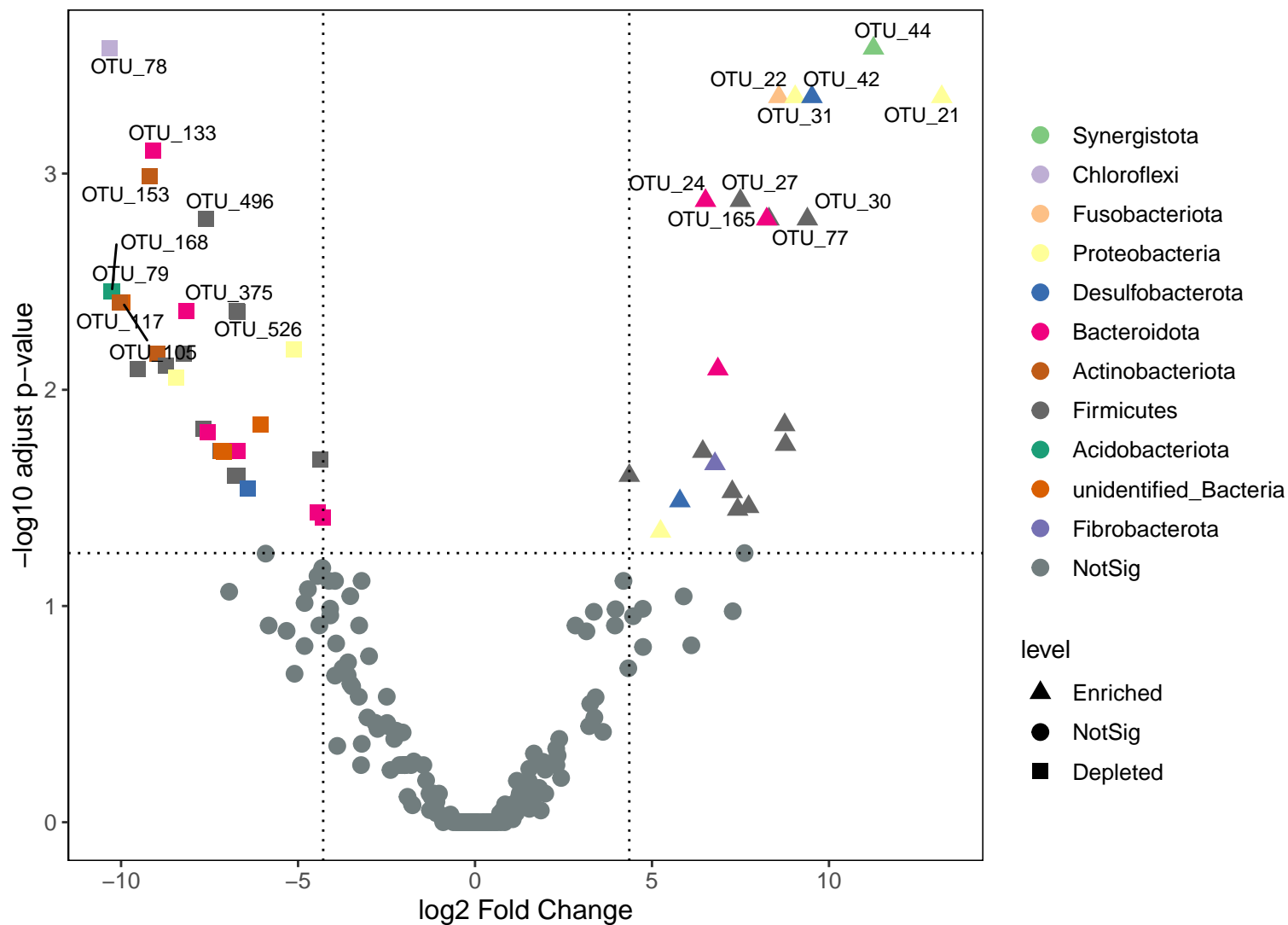

Supplement: Supplementary file 1 [file Data_Sheet_1.ZIP › Supplement Figure Volcano/M10 vs M9 _Volcano plot.pdf]

# M11 vs M6

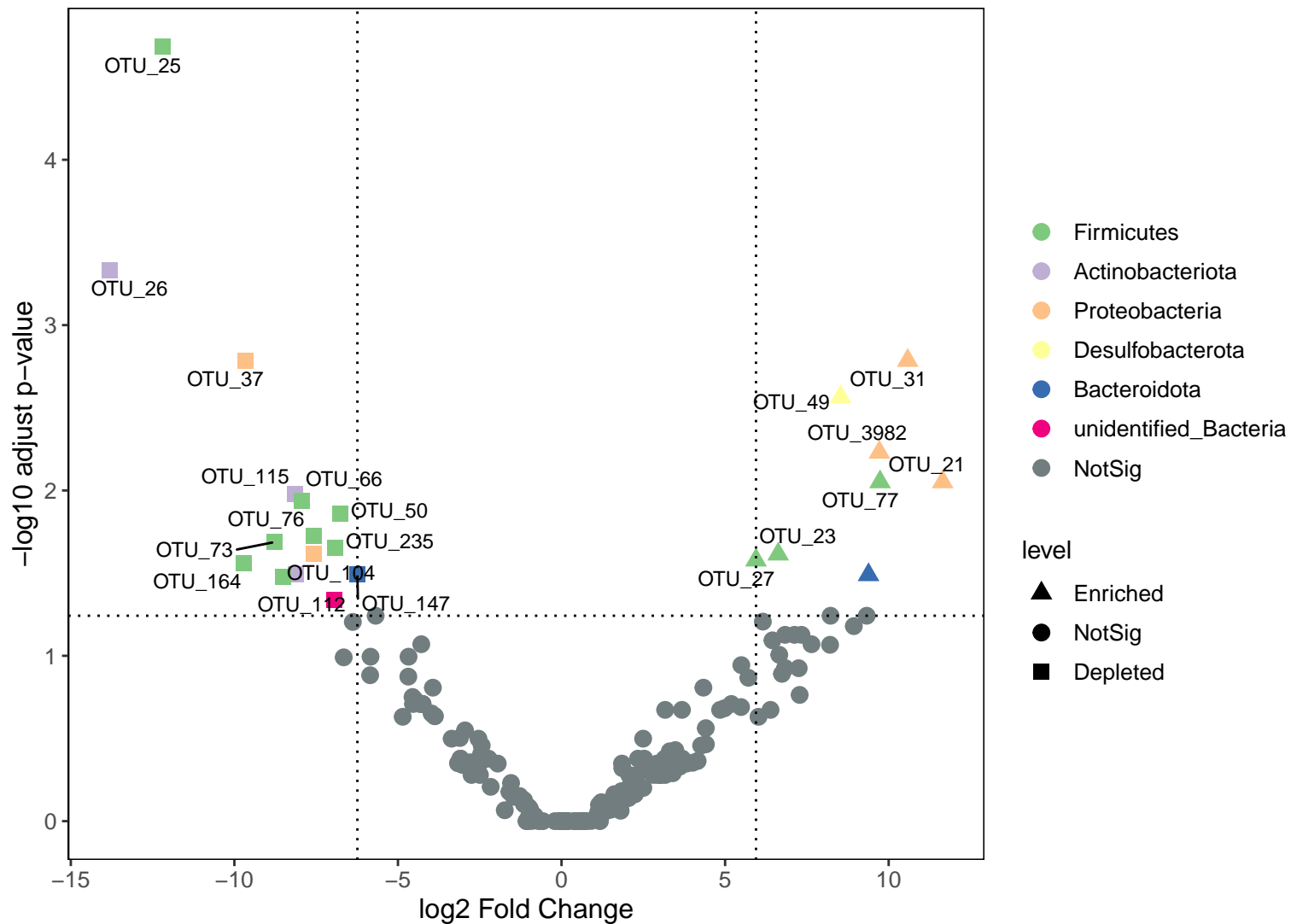

Supplement: Supplementary file 1 [file Data_Sheet_1.ZIP › Supplement Figure Volcano/M11 vs M5_Volcano plot.pdf]

M11 vs M6

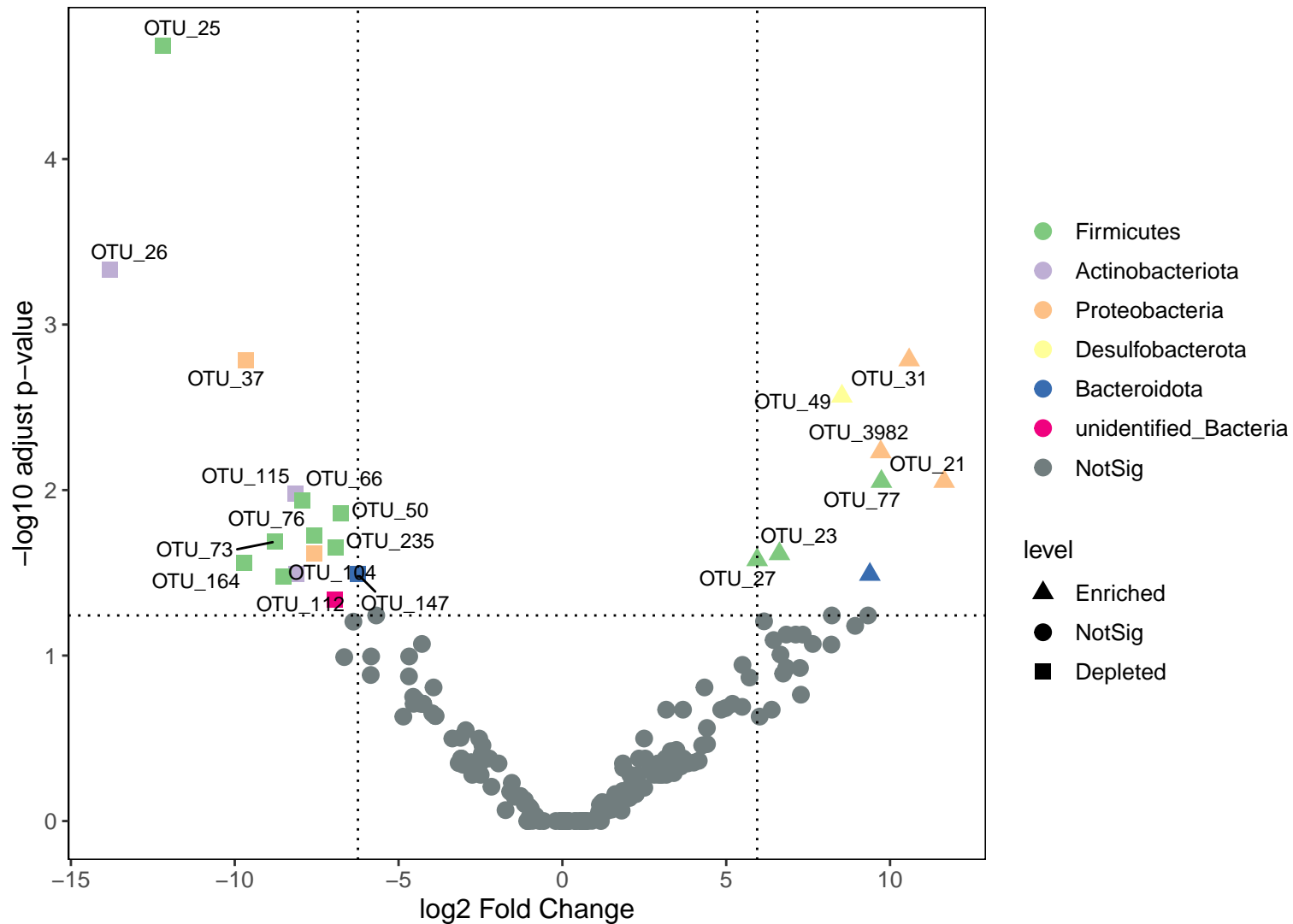

Supplement: Supplementary file 1 [file Data_Sheet_1.ZIP › Supplement Figure Volcano/M11 vs M6 _Volcano plot.pdf]

M11 vs M7

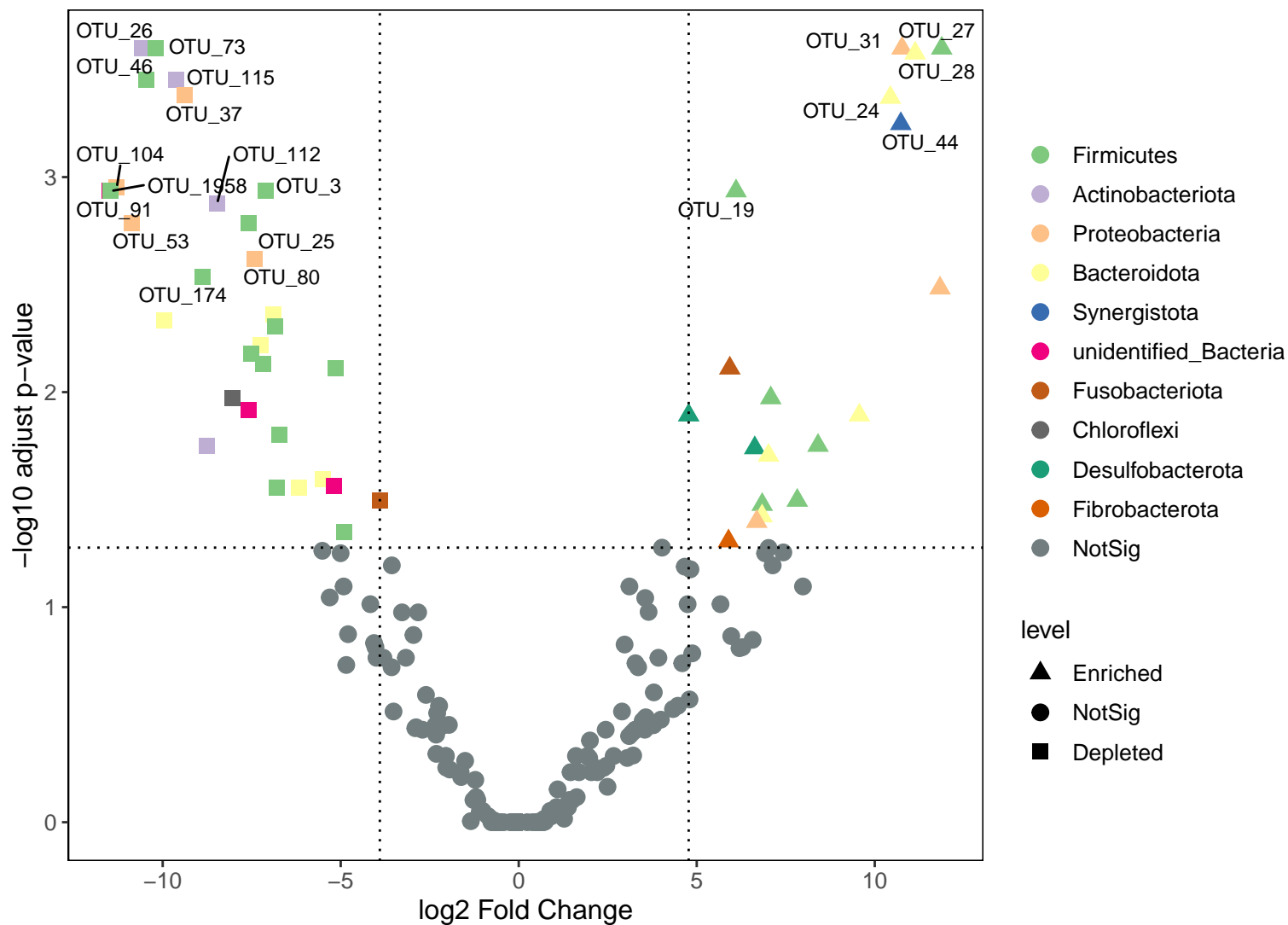

Supplement: Supplementary file 1 [file Data_Sheet_1.ZIP › Supplement Figure Volcano/M11 vs M7 _Volcano plot.pdf]

M11 vs M8

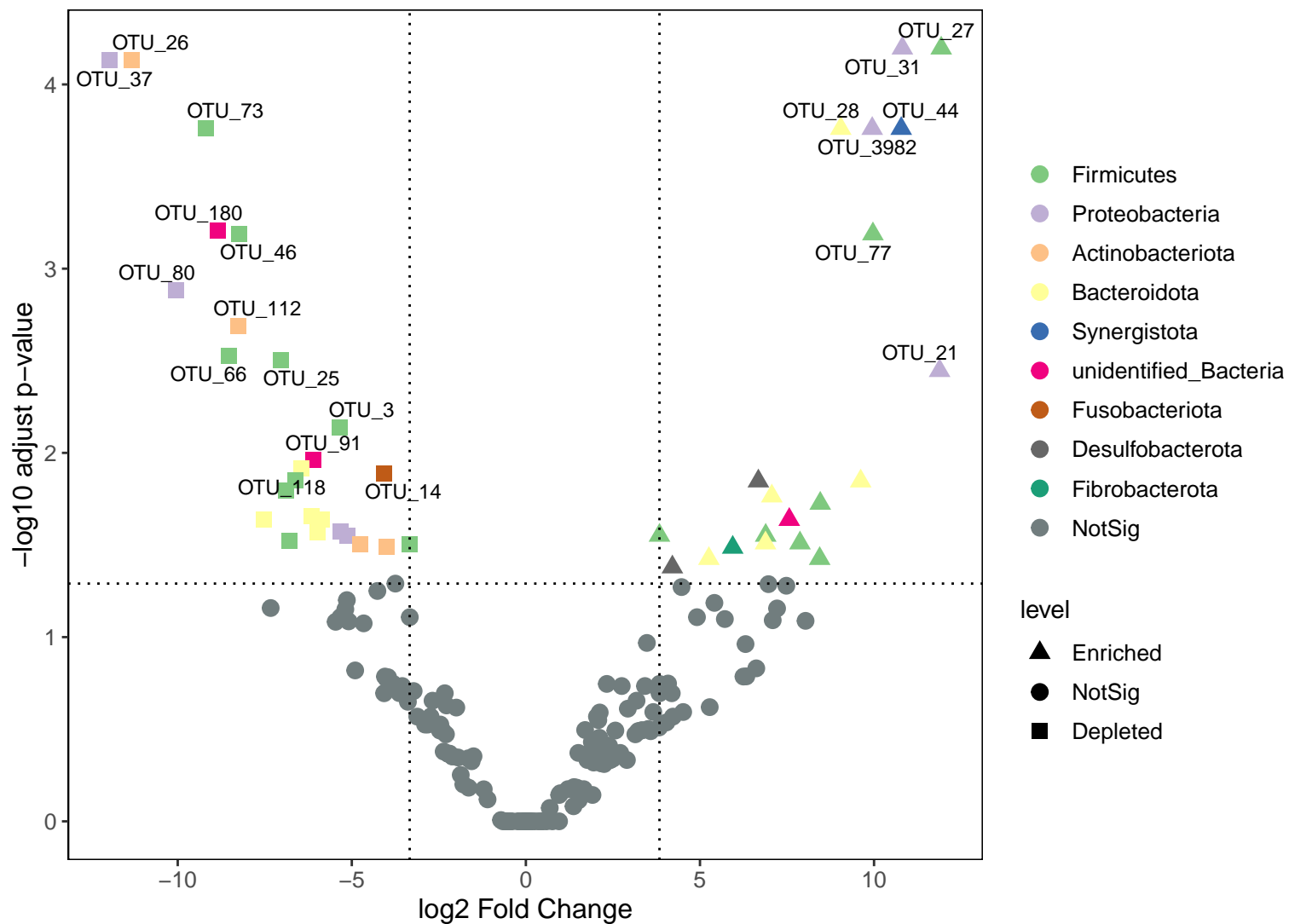

Supplement: Supplementary file 1 [file Data_Sheet_1.ZIP › Supplement Figure Volcano/M11 vs M8 _Volcano plot.pdf]

M11 vs M9

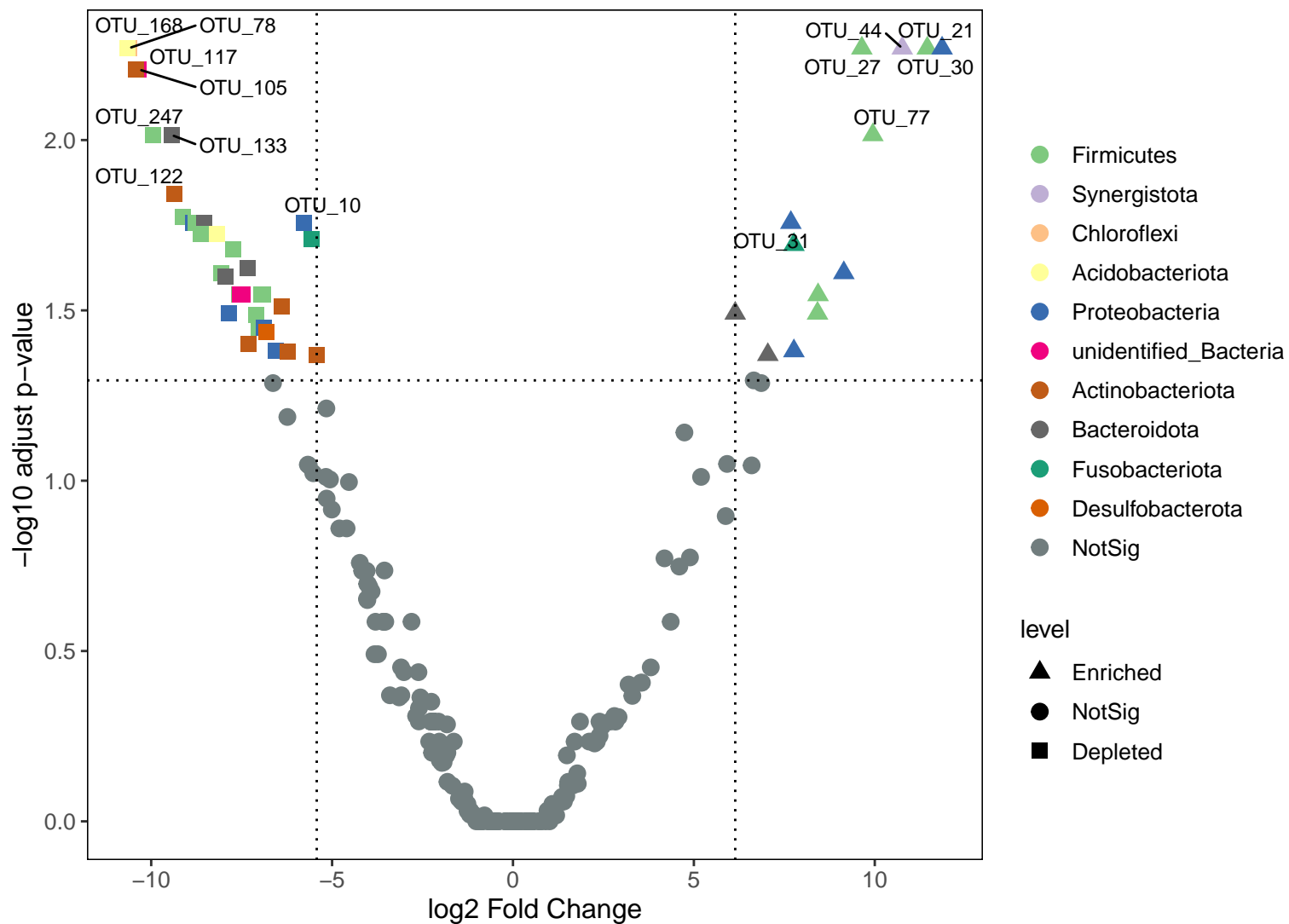

Supplement: Supplementary file 1 [file Data_Sheet_1.ZIP › Supplement Figure Volcano/M11 vs M9 _Volcano plot.pdf]

# M12 vs M10

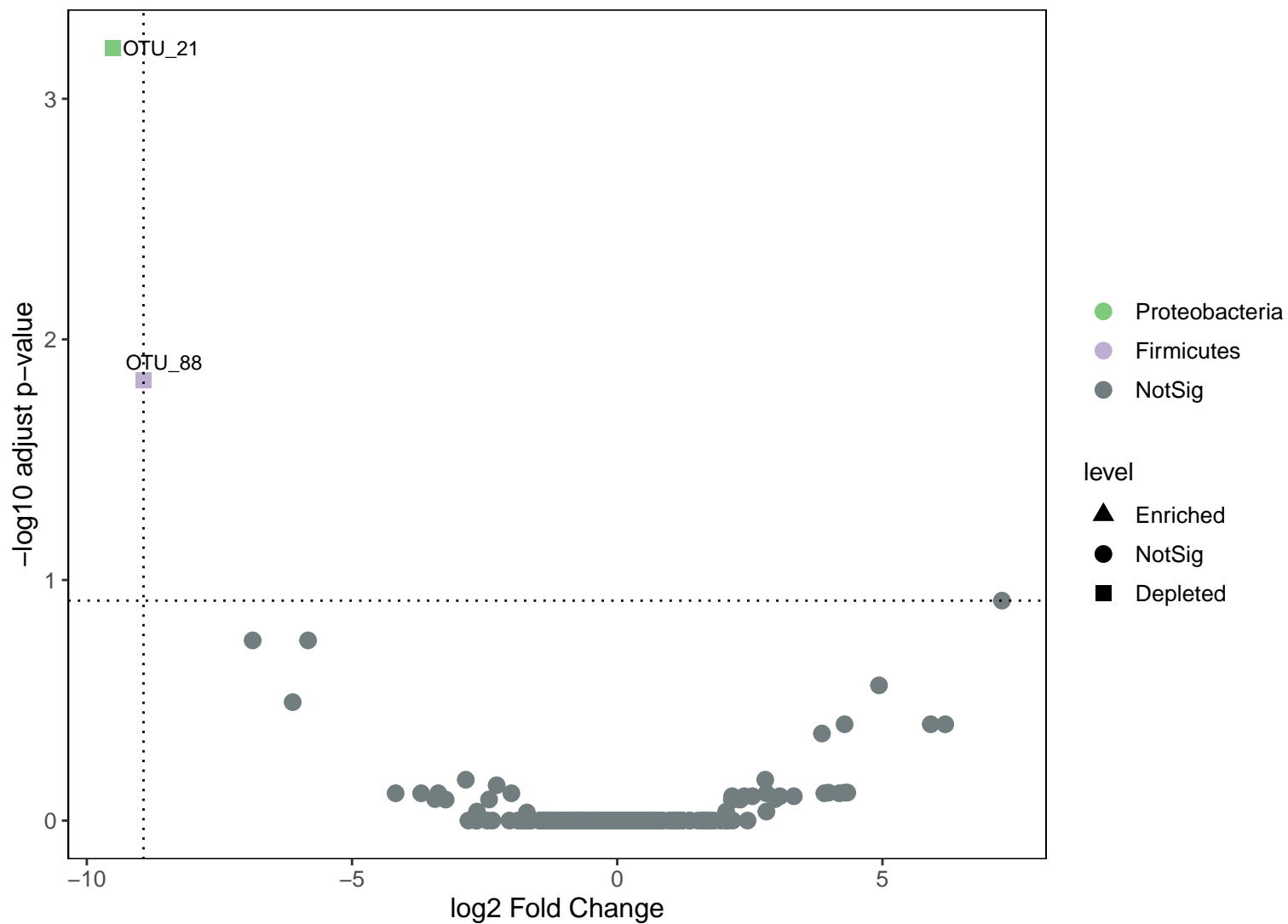

Supplement: Supplementary file 1 [file Data_Sheet_1.ZIP › Supplement Figure Volcano/M12 vs M10 _Volcano plot.pdf]

# M12 vs M11

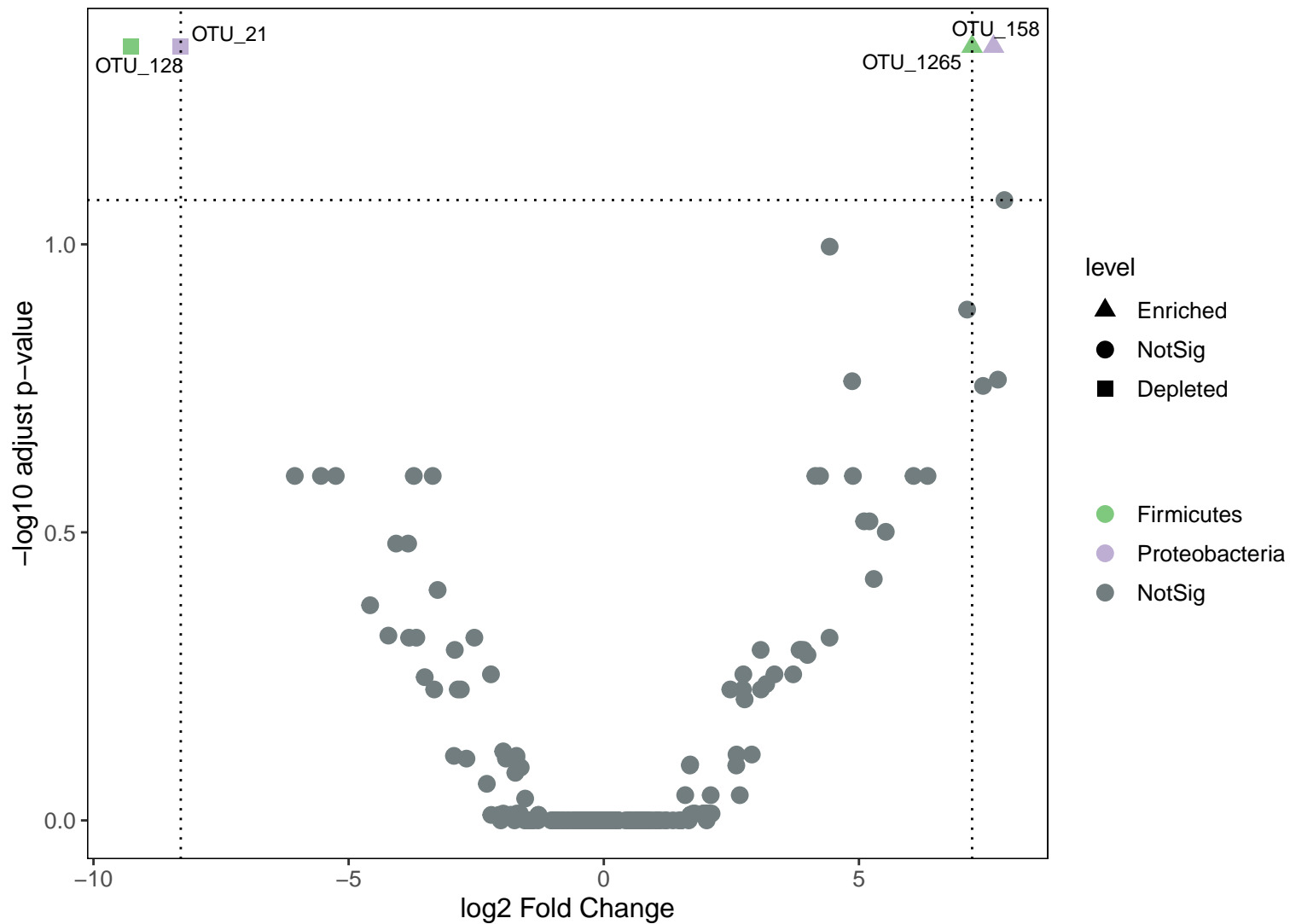

Supplement: Supplementary file 1 [file Data_Sheet_1.ZIP › Supplement Figure Volcano/M12 vs M11 _Volcano plot.pdf]

# M12 vs M5

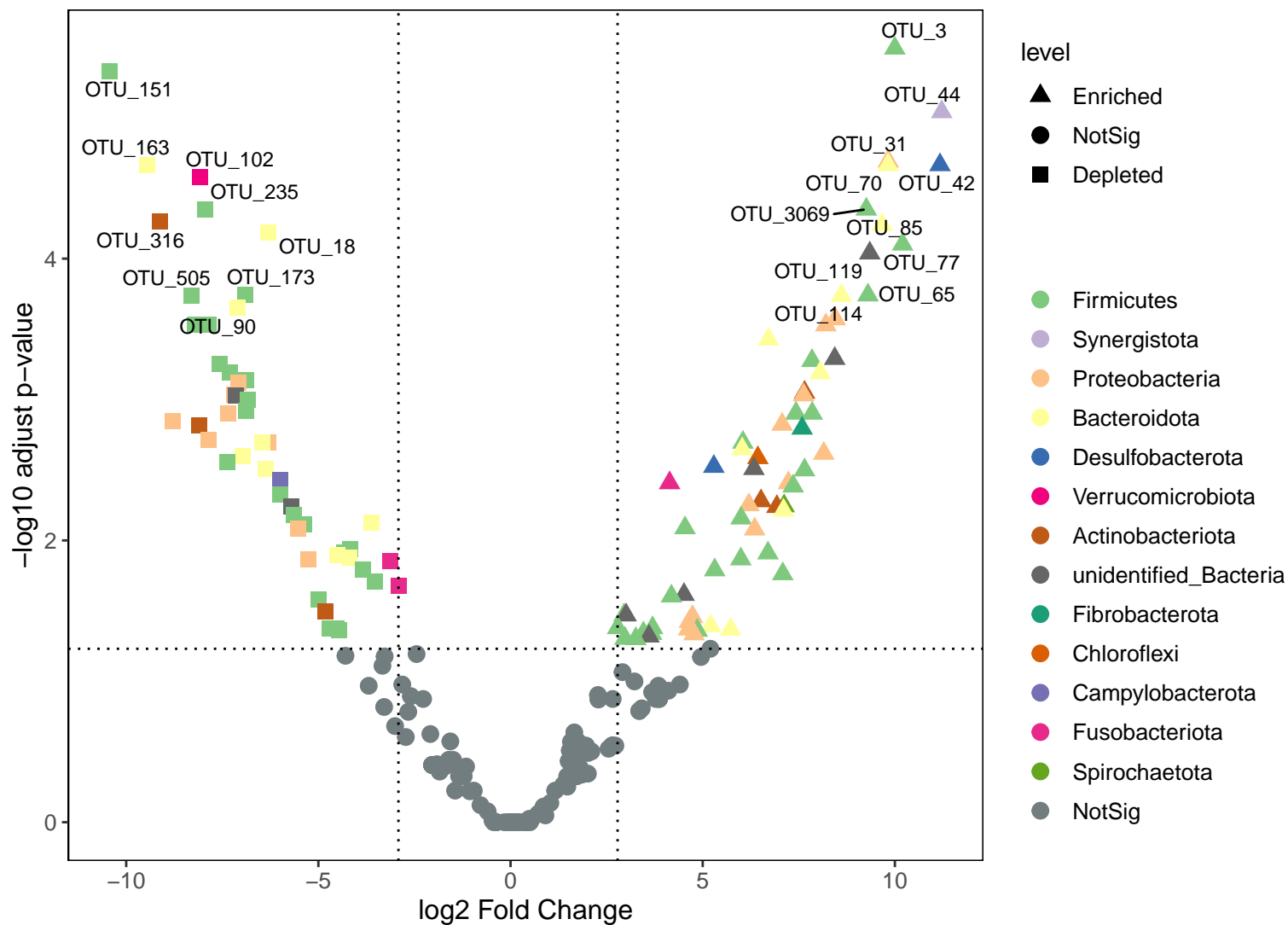

Supplement: Supplementary file 1 [file Data_Sheet_1.ZIP › Supplement Figure Volcano/M12 vs M5_Volcano plot.pdf]

# M12 vs M6

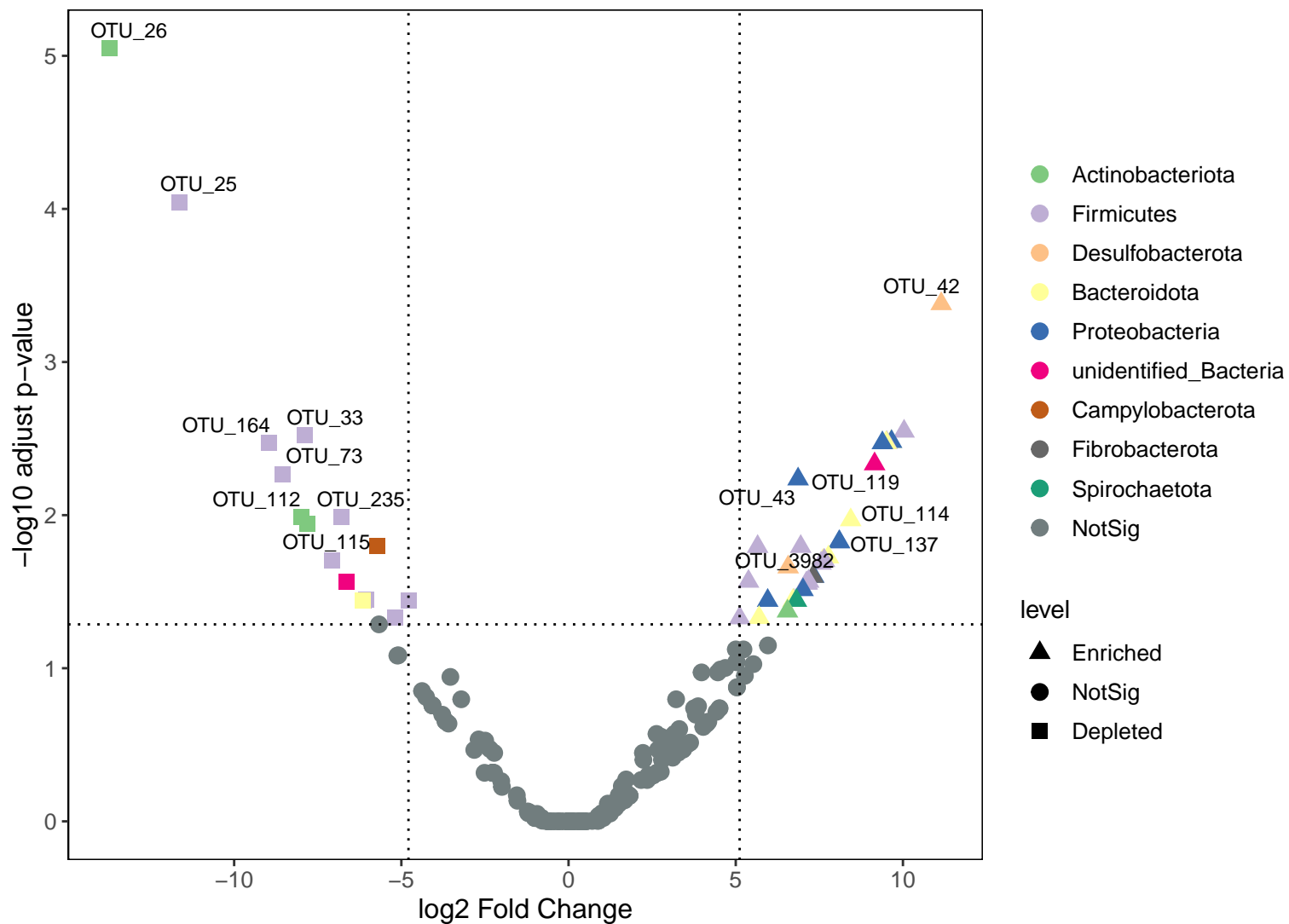

Supplement: Supplementary file 1 [file Data_Sheet_1.ZIP › Supplement Figure Volcano/M12 vs M6 _Volcano plot.pdf]

# M12 vs M7

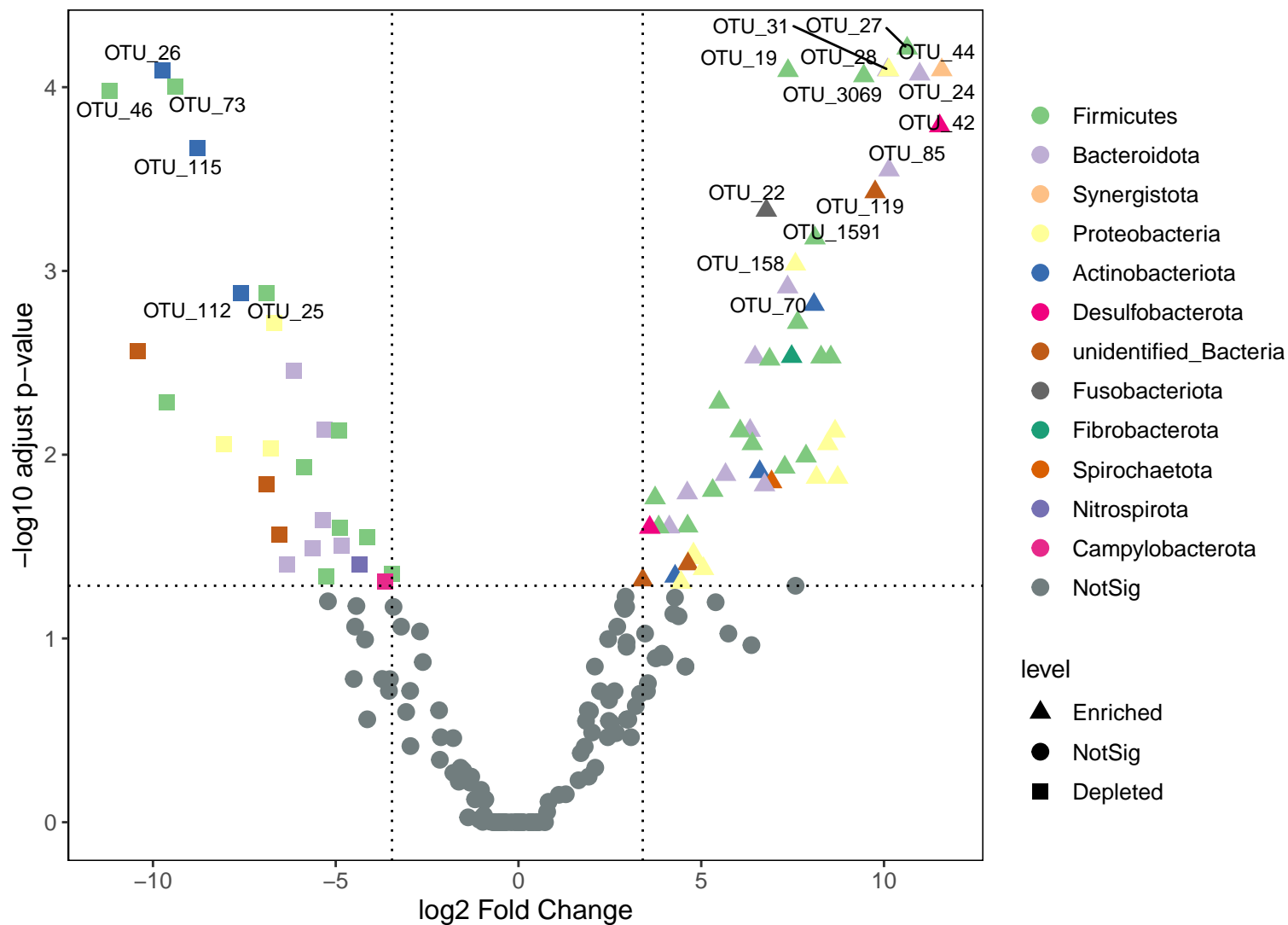

Supplement: Supplementary file 1 [file Data_Sheet_1.ZIP › Supplement Figure Volcano/M12 vs M7 _Volcano plot.pdf]

# M12 vs M8

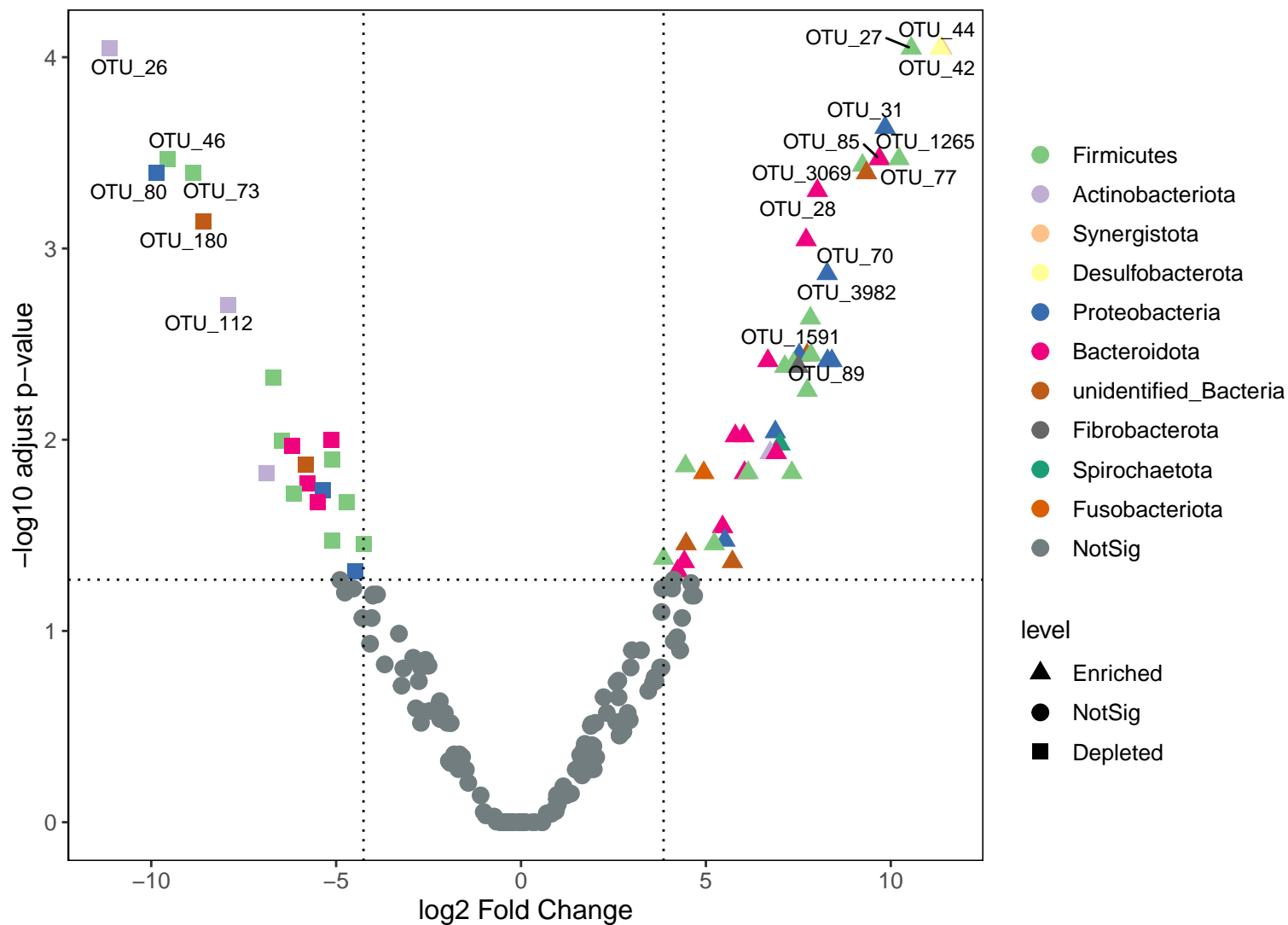

Supplement: Supplementary file 1 [file Data_Sheet_1.ZIP › Supplement Figure Volcano/M12 vs M8 _Volcano plot.pdf]

# M12 vs M9

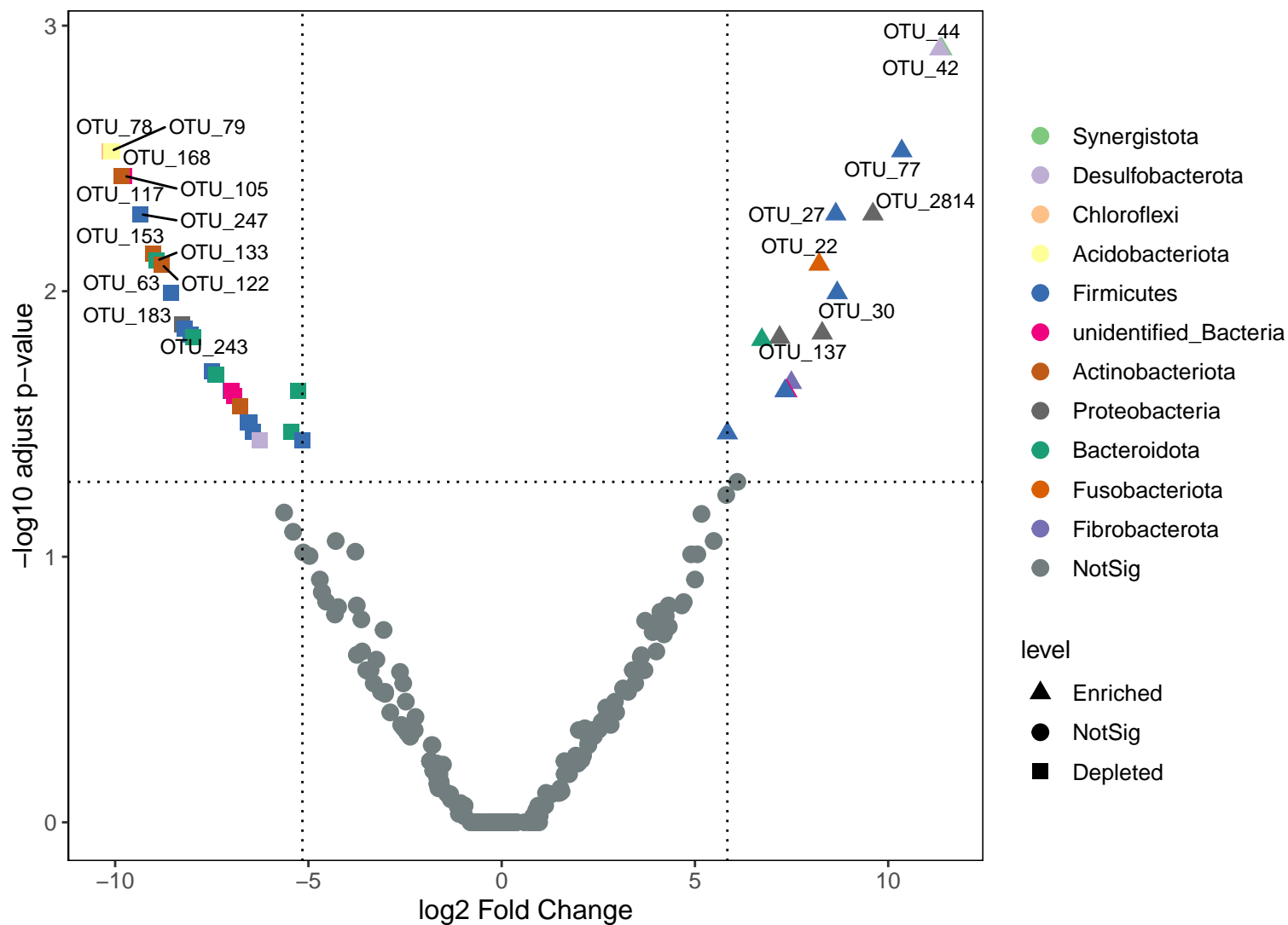

Supplement: Supplementary file 1 [file Data_Sheet_1.ZIP › Supplement Figure Volcano/M12 vs M9 _Volcano plot.pdf]

# M6 vs M5

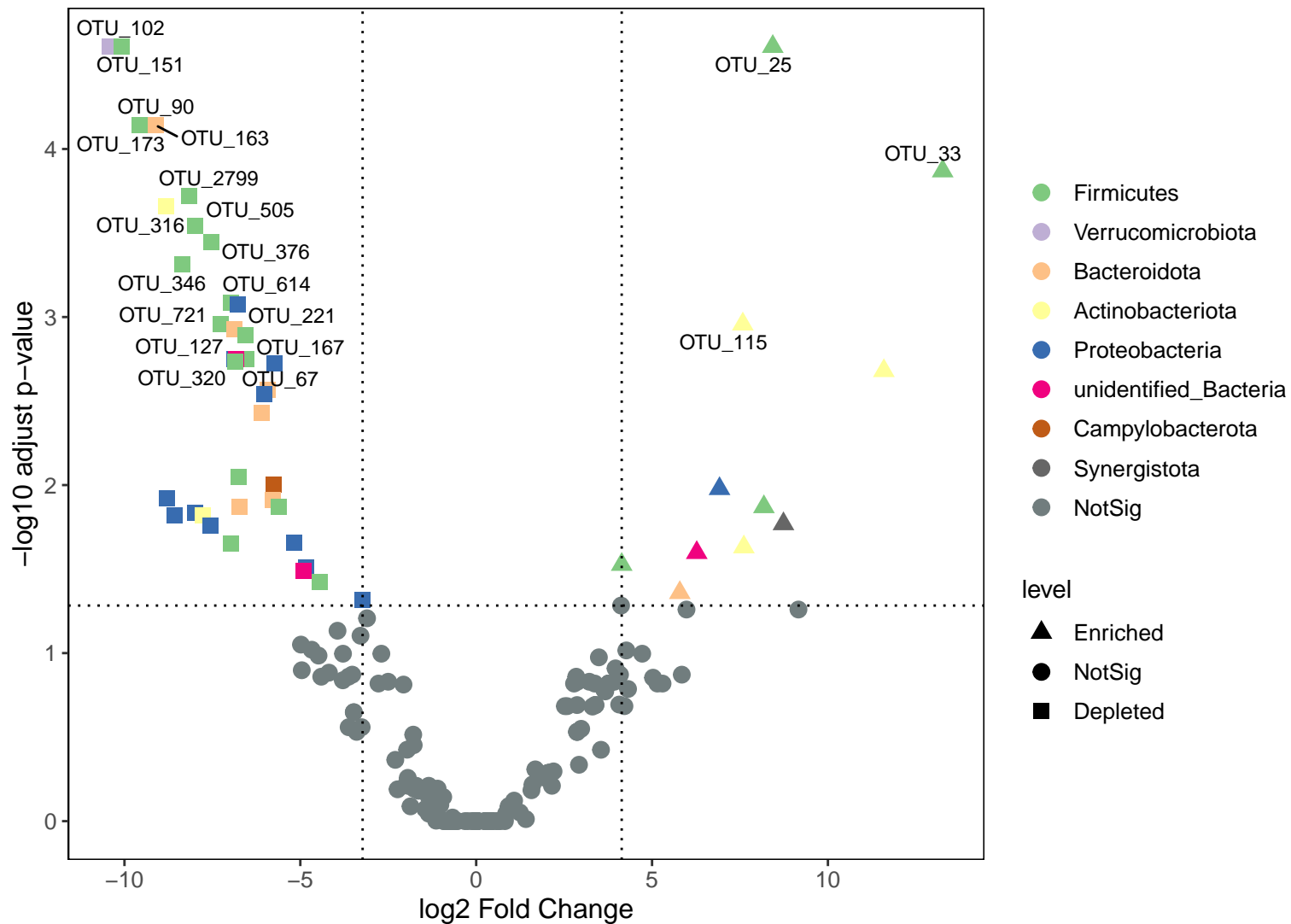

Supplement: Supplementary file 1 [file Data_Sheet_1.ZIP › Supplement Figure Volcano/M6 vs M5_Volcano plot.pdf]

# M7 vs M5

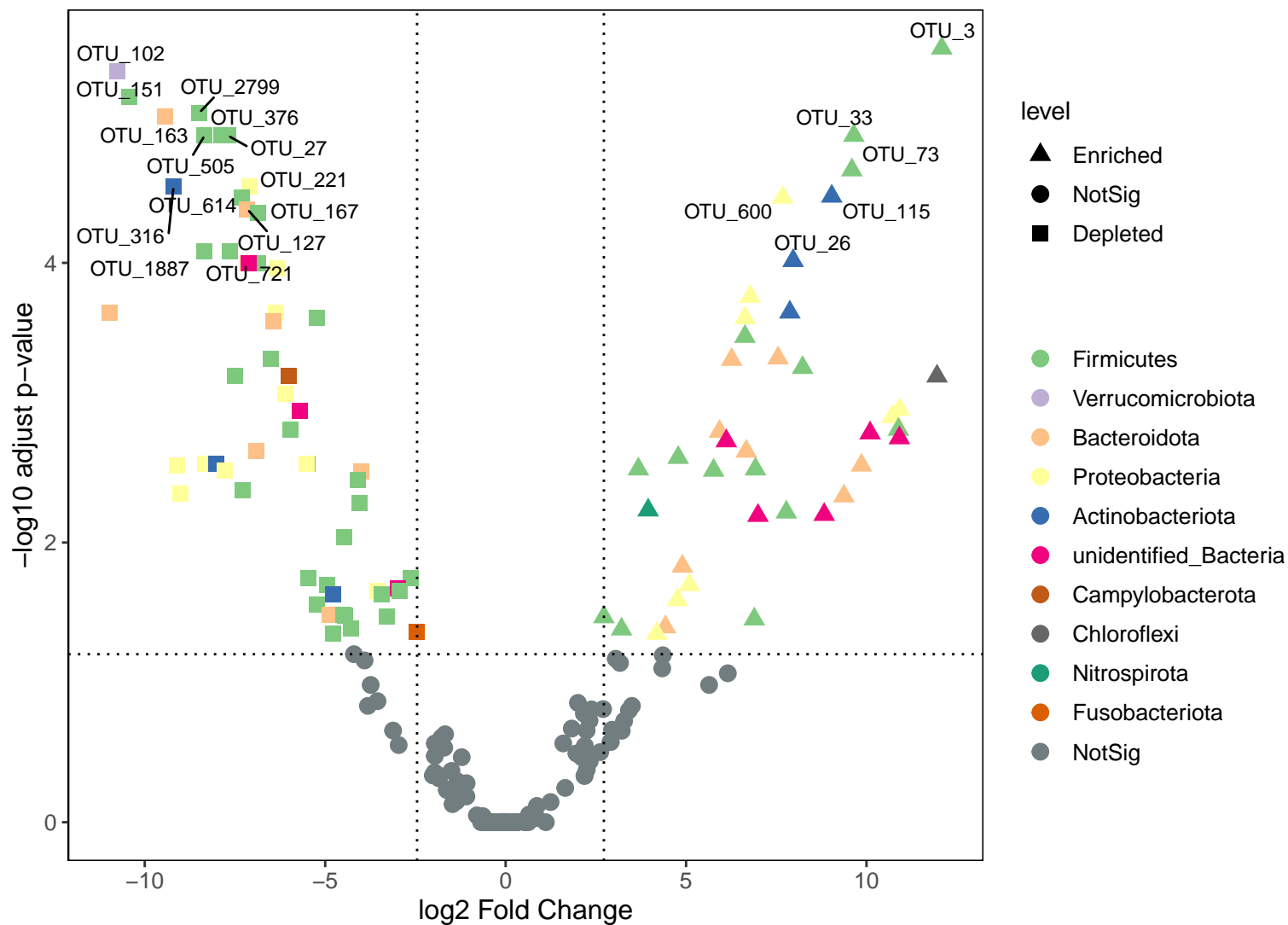

Supplement: Supplementary file 1 [file Data_Sheet_1.ZIP › Supplement Figure Volcano/M7 vs M5_Volcano plot.pdf]

# M7 vs M6

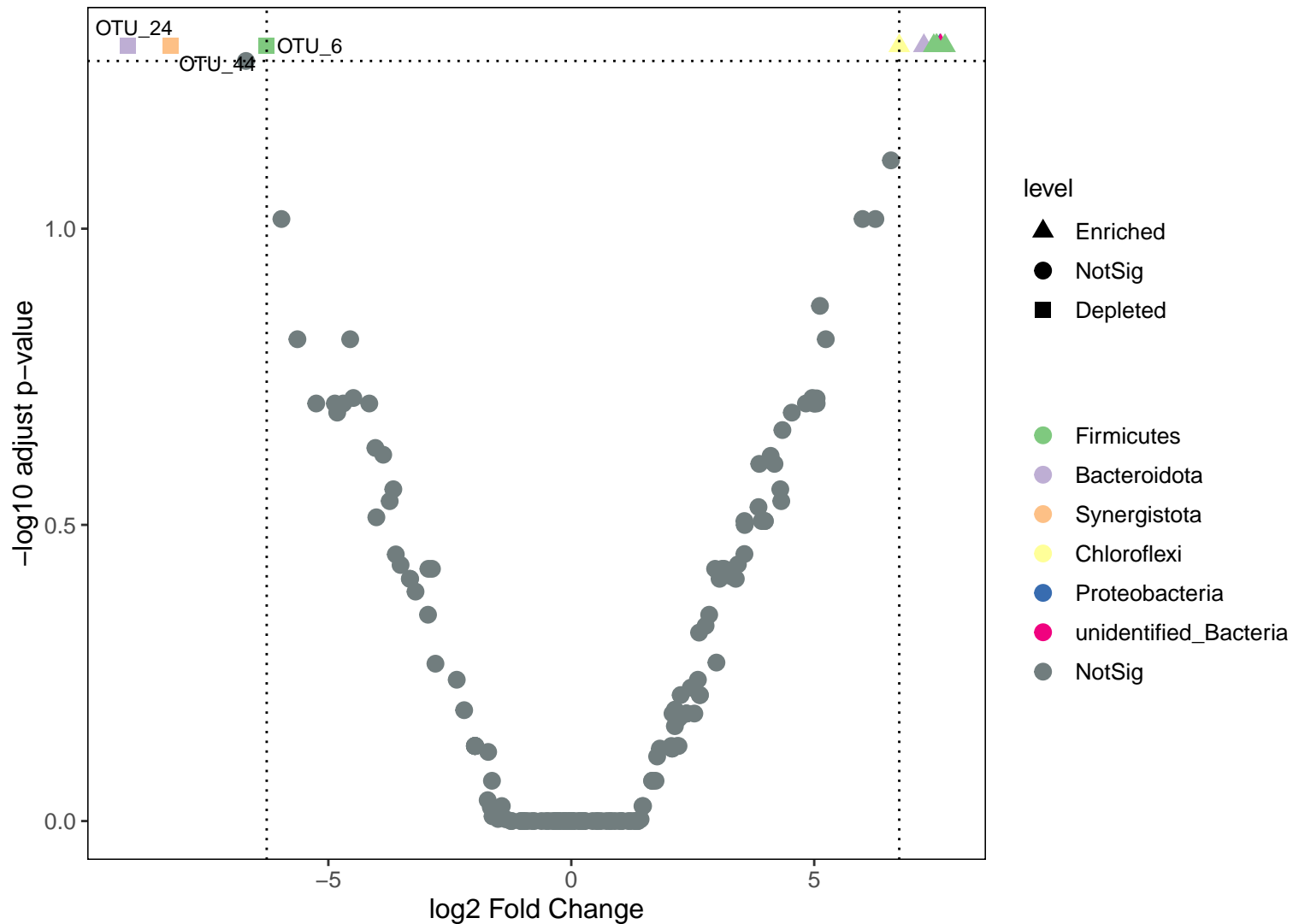

Supplement: Supplementary file 1 [file Data_Sheet_1.ZIP › Supplement Figure Volcano/M7 vs M6 _Volcano plot.pdf]

# M8 vs M5

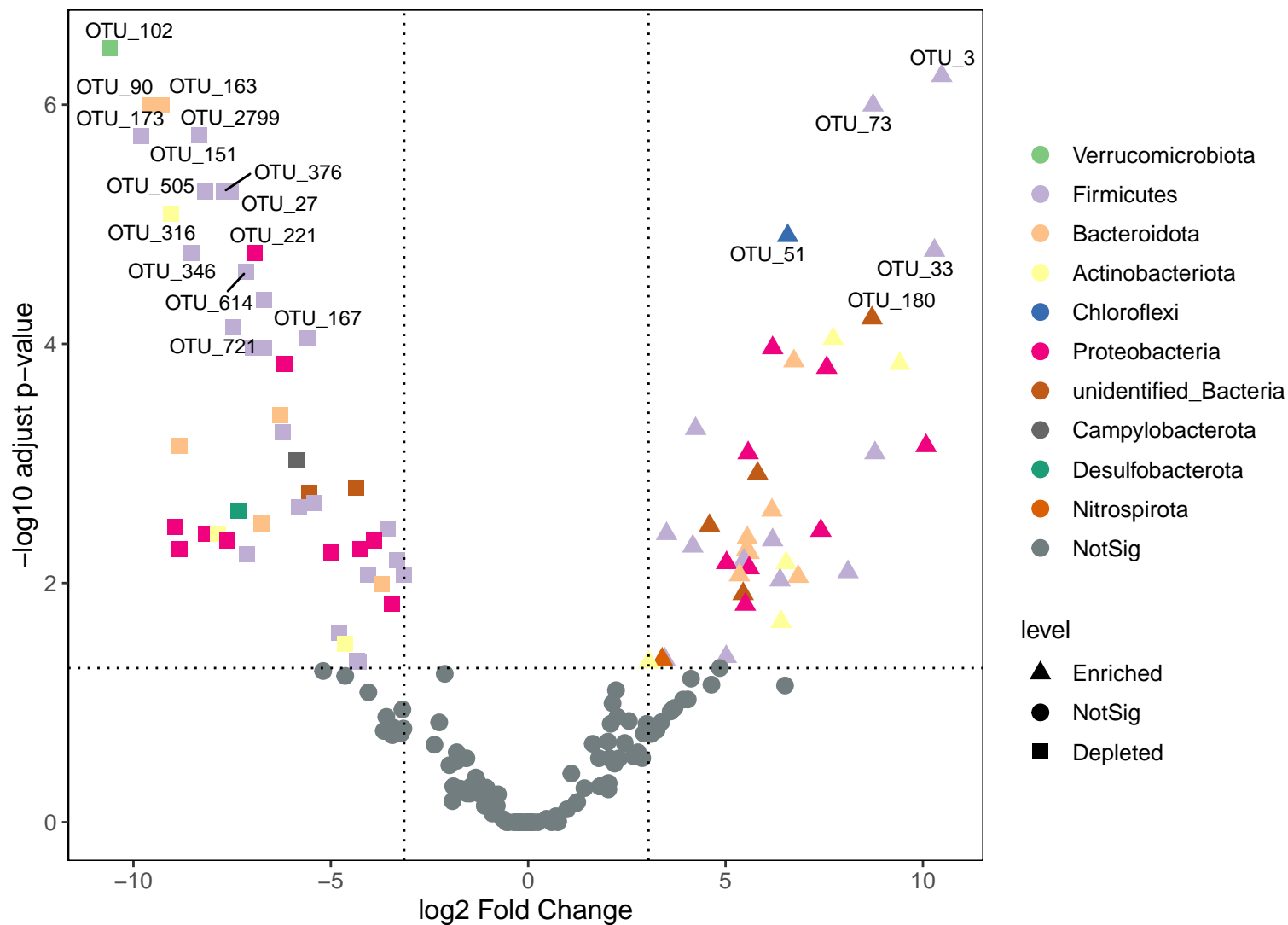

Supplement: Supplementary file 1 [file Data_Sheet_1.ZIP › Supplement Figure Volcano/M8 vs M5_Volcano plot.pdf]

# M9 vs M5

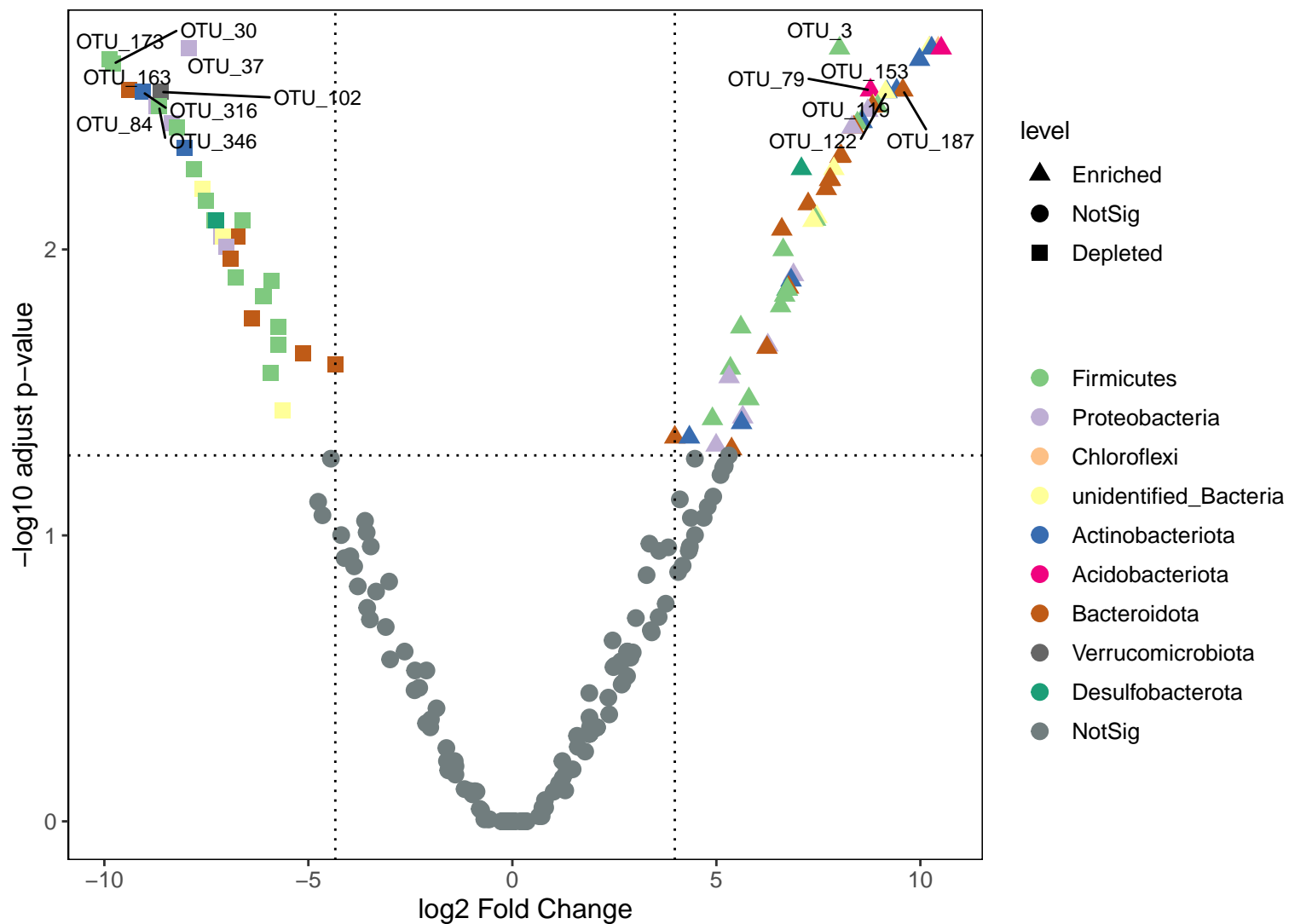

Supplement: Supplementary file 1 [file Data_Sheet_1.ZIP › Supplement Figure Volcano/M9 vs M5_Volcano plot.pdf]

# M9 vs M6

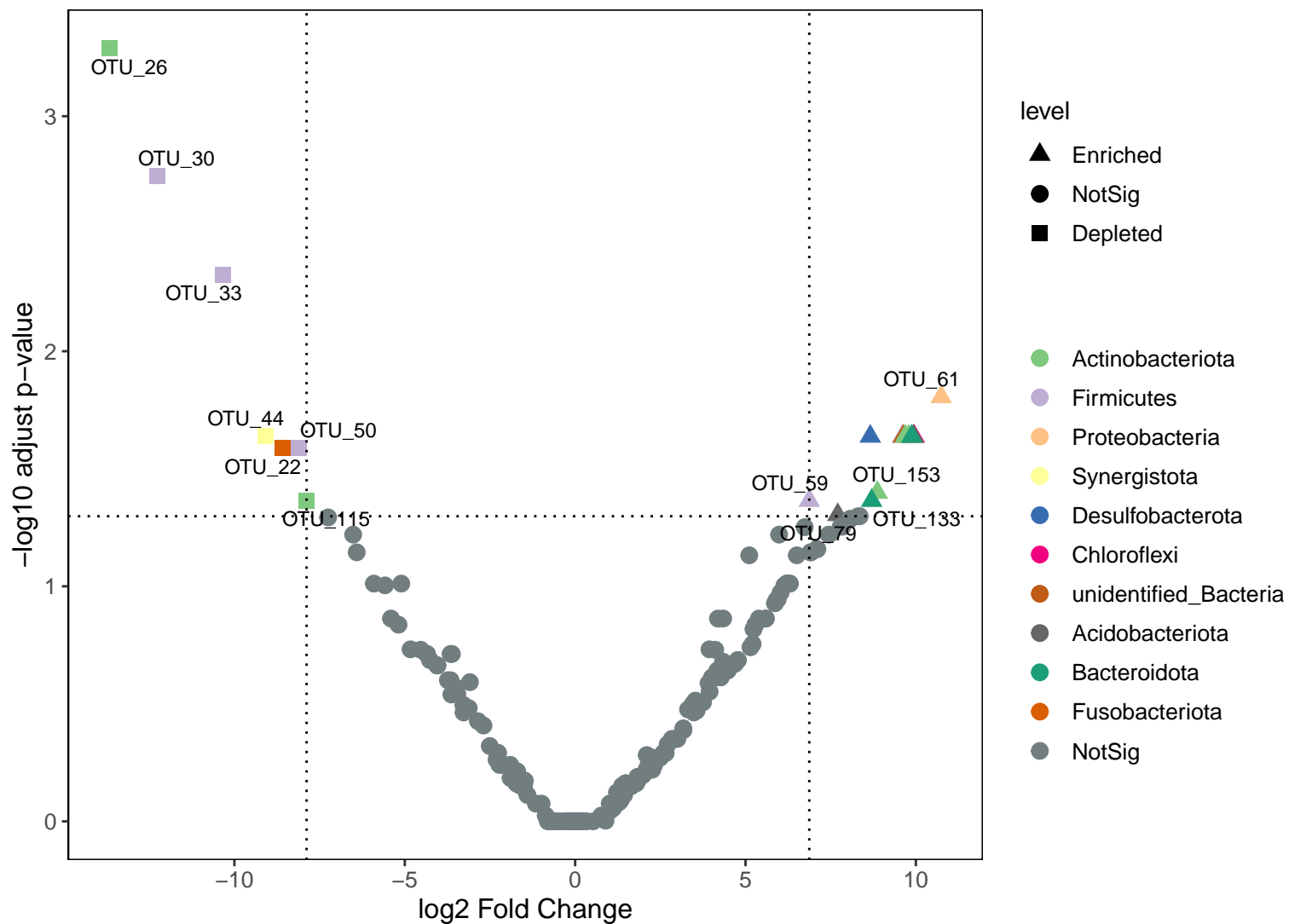

Supplement: Supplementary file 1 [file Data_Sheet_1.ZIP › Supplement Figure Volcano/M9 vs M6 _Volcano plot.pdf]

# M9 vs M7

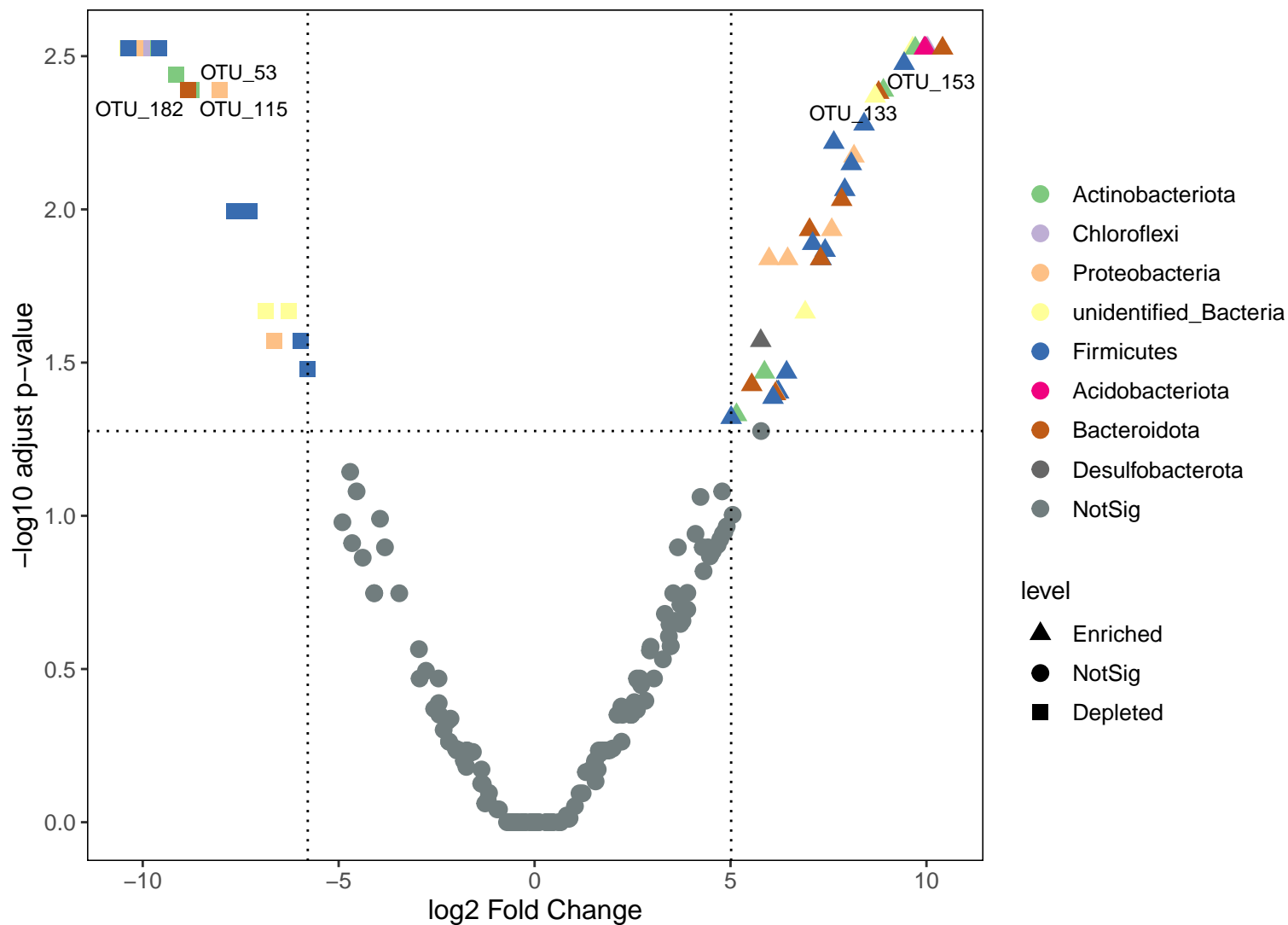

Supplement: Supplementary file 1 [file Data_Sheet_1.ZIP › Supplement Figure Volcano/M9 vs M7 _Volcano plot.pdf]

# M9 vs M8

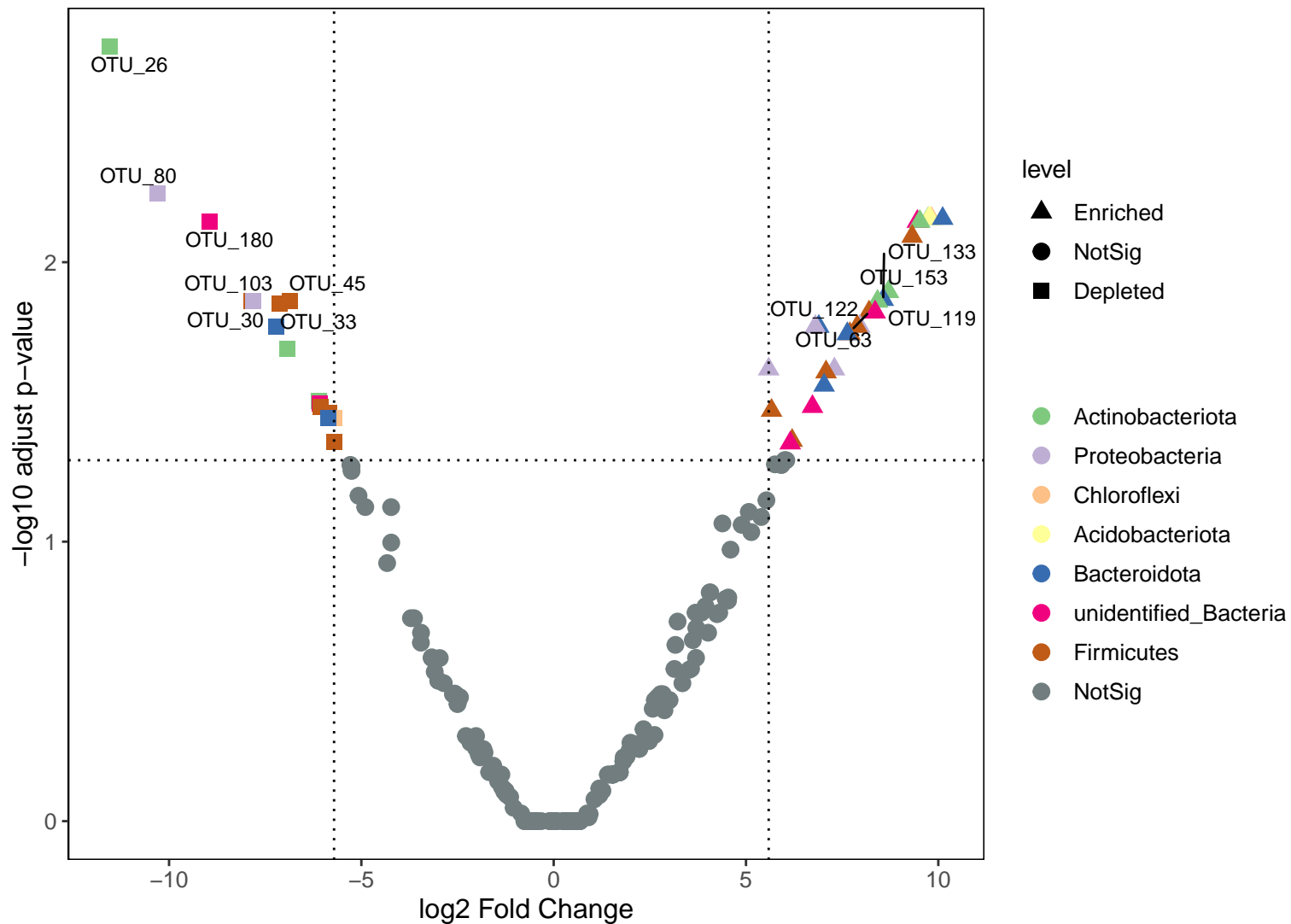

Supplement: Supplementary file 1 [file Data_Sheet_1.ZIP › Supplement Figure Volcano/M9 vs M8 _Volcano plot.pdf]

# subadult vs M12

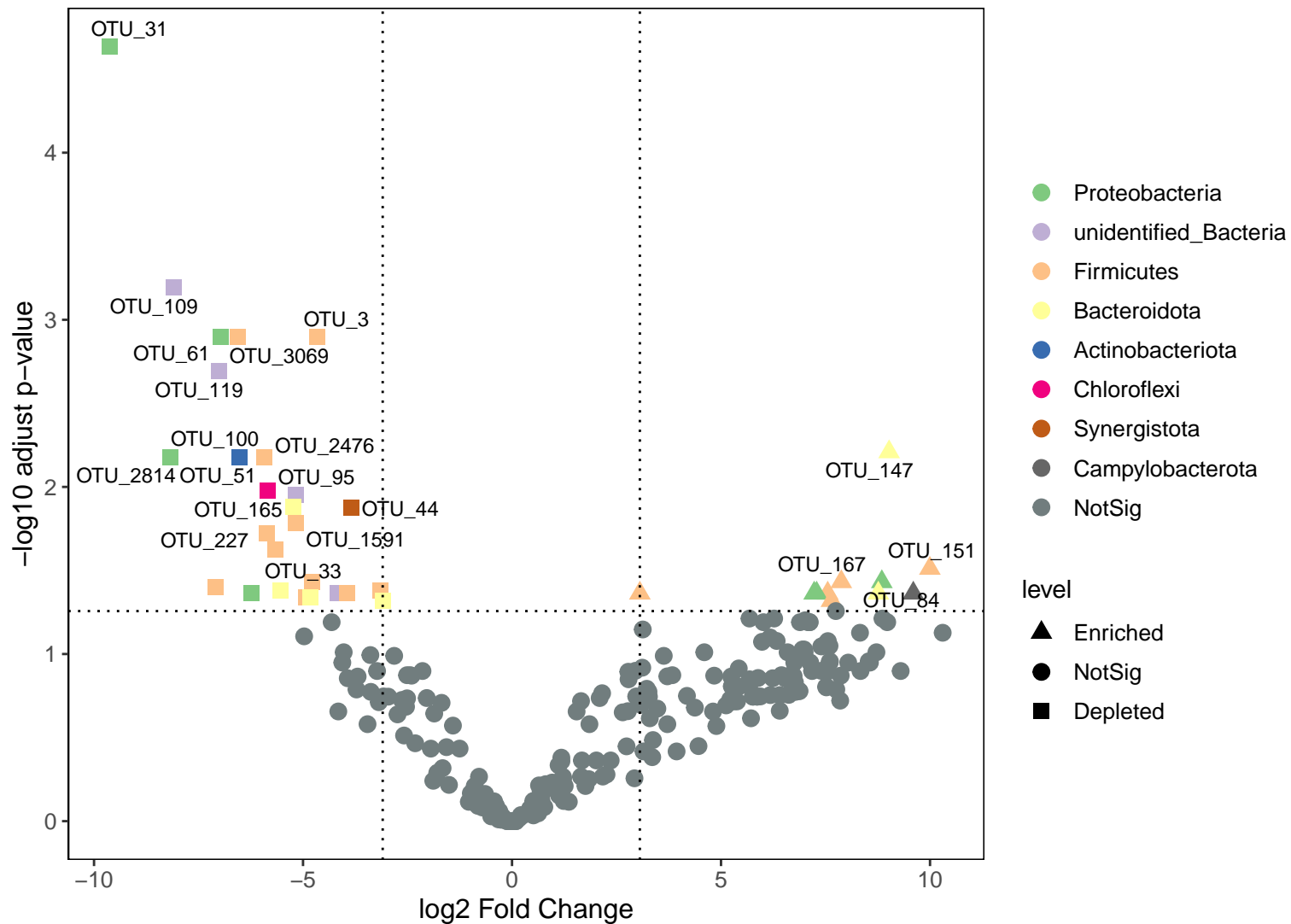

Supplement: Supplementary file 1 [file Data_Sheet_1.ZIP › Supplement Figure Volcano/subadult vs M12 _Volcano plot.pdf]
