# Supplementary material for: Developmental stage variation in the gut microbiome of South China tigers
Source: Front Microbiol. 2022 Nov 9;13:962614. doi: 10.3389/fmicb.2022.962614 (PMC9682017; doi:10.3389/fmicb.2022.962614)
Supplement: Supplementary Figure 1 — Volcano grams of other subgroups of intestinal bacteria of the South China tiger. [file Data_Sheet_2.pdf]

## Supplemental Information for:

# Developmental stage variation in the gut microbiome of South China tigers

**Xianfu Zhang[1#], Yanxin Liao[1#], Tao Qin[2], Jinghua Ma[3], Jianxun Liu[3], Jianqiang Zou[3], Haijun Huang[3], Xiaojun Zhong[1*], Menghua Yang[1*]**

1. College of Animal Science and Technology, College of Veterinary Medicine, Zhejiang A & F University, Key Laboratory of Applied Technology on Green-Eco-Healthy Animal Husbandry of Zhejiang Province, Zhejiang Provincial Engineering Laboratory for Animal Health Inspection & Internet Technology, Hangzhou, China
2. State Key Laboratory of Subtropical Silviculture, College of Forestry and Biotechnology, Zhejiang A&F University, Hangzhou, China
3. Hangzhou Safari Park, Hangzhou, China

**Supplemental Table S1. Tigers used in this study**

| Names | Age | Group | Sub-Species | Sex | Sampling data | Pedigree information |
|---|---|---|---|---|---|---|
| AA1 | 5 months | Cub (M5) | Southern China Tiger | Female | 2021.01.21 | 900038000425514 |
| LL1 | 5 months | Cub (M5) | Southern China Tiger | Female | 2021.01.20 | 900038000425535 |
| GG1 | 5 months | Cub (M5) | Southern China Tiger | Female | 2021.01.19 | 900038000425687 |
| AA2 | 6 months | Cub (M6) | Southern China Tiger | Female | 2021.02.28 | 900038000425514 |
| LL2 | 6 months | Cub (M6) | Southern China Tiger | Female | 2021.02.28 | 900038000425535 |
| GG2 | 6 months | Cub (M6) | Southern China Tiger | Female | 2021.02.28 | 900038000425687 |
| AA3 | 7 months | Cub (M7) | Southern China Tiger | Female | 2021.04.04 | 900038000425514 |
| LL3 | 7 months | Cub (M7) | Southern China Tiger | Female | 2021.04.03 | 900038000425535 |
| GG3 | 7 months | Cub (M7) | Southern China Tiger | Female | 2021.04.02 | 900038000425687 |
| AA4 | 8 months | Cub (M8) | Southern China Tiger | Female | 2021.04.25 | 900038000425514 |
| LL4 | 8 months | Cub (M8) | Southern China Tiger | Female | 2021.04.24 | 900038000425535 |
| GG4 | 8 months | Cub (M8) | Southern China Tiger | Female | 2021.04.25 | 900038000425687 |
| AA5 | 9 months | Cub (M9) | Southern China Tiger | Female | 2021.05.26 | 900038000425514 |
| LL5 | 9 months | Cub (M9) | Southern China Tiger | Female | 2021.05.26 | 900038000425535 |
| GG5 | 9 months | Cub (M9) | Southern China Tiger | Female | 2021.05.27 | 900038000425687 |
| AA6 | 10 months | Cub (M10) | Southern China Tiger | Female | 2021.06.28 | 900038000425514 |
| LL6 | 10 months | Cub (M10) | Southern China Tiger | Female | 2021.06.27 | 900038000425535 |
| GG6 | 10 months | Cub (M10) | Southern China Tiger | Female | 2021.06.27 | 900038000425687 |
| AA7 | 11 months | Cub (M11) | Southern China Tiger | Female | 2021.07.27 | 900038000425514 |
| LL7 | 11 months | Cub (M11) | Southern China Tiger | Female | 2021.07.27 | 900038000425535 |
| GG7 | 11 months | Cub (M11) | Southern China Tiger | Female | 2021.07.27 | 900038000425687 |
| AA8 | 1 year | Cub (M12) | Southern China Tiger | Female | 2021.08.25 | 900038000425514 |
| LL8 | 1 year | Cub (M12) | Southern China Tiger | Female | 2021.08.23 | 900038000425535 |
| GG8 | 1 year | Cub (M12) | Southern China Tiger | Female | 2021.08.24 | 900038000425687 |

| | | | | | | |
|---|---|---|---|---|---|---|
| WZ | 3.5 years | Subadult | Southern China Tiger | Male | 2020.12.28 | 900038000425660 |
| TT | 3 years | Subadult | Southern China Tiger | Male | 2020.12.28 | 900038000425768 |
| YY | 3 years | Subadult | Southern China Tiger | Male | 2021.01.05 | 900038000425868 |
| MAN | 3 years | Subadult | Southern China Tiger | Female | 2020.12.30 | 900038000425818 |
| MEI | 2.5 years | Subadult | Southern China Tiger | Female | 2021.01.17 | 900038000425485 |
| JJ | 2.5 years | Subadult | Southern China Tiger | Male | 2021.01.11 | 900038000425724 |
| QQ | 2.5 years | Subadult | Southern China Tiger | Male | 2021.01.12 | 900038000425538 |
| MAO | 2.5 years | Subadult | Southern China Tiger | Male | 2020.12.26 | 900038000425832 |
| MW | 6 years | Adult (SC) | Southern China Tiger | Female | 2020.12.26 | 900038000077808 |
| ER | 6.5 years | Adult (SC) | Southern China Tiger | Female | 2020.12.26 | 900038000077789 |
| DB | 8 years | Adult (SC) | Southern China Tiger | Female | 2020.12.29 | 900038000383758 |
| WEI | 8.5 years | Adult (SC) | Southern China Tiger | Male | 2020.12.28 | 900038000219014 |
| LONG | 11 years | Adult (SC) | Southern China Tiger | Male | 2020.12.26 | 900038000035266 |
| M5 | 8 years | Adult (Bg) | Bengal Tiger | Female | 2021.01.03 | 900038000425739 |
| M6 | 13 years | Adult (Bg) | Bengal Tiger | Male | 2021.01.04 | 900038000425784 |
| M7 | 6-7 years | Adult (Bg) | Bengal Tiger | Female | 2021.01.10 | 900038000425849 |
| M8 | 6-8 years | Adult (Bg) | Bengal Tiger | Female | 2021.01.11 | 900038000425823 |
| M9 | 6-9 years | Adult (Bg) | Bengal Tiger | Male | 2021.01.11 | 900038000425822 |
| D2 | 5 years | Adult (Am) | Amur Tiger | Male | 2020.12.28 | 900038000425504 |
| D4 | 6 years | Adult (Am) | Amur Tiger | Female | 2020.12.28 | 900038000425775 |
| D5 | 5 years | Adult (Am) | Amur Tiger | Male | 2020.12.28 | 900038000075028 |
| D3 | Old | Adult (Am) | Amur Tiger | Female | 2020.12.28 | 900038000002028 |
| D7 | Old | Adult (Am) | Amur Tiger | Female | 2020.12.28 | 900038000018200 |

**Supplemental Table S2. Food provided to tigers.**

| Groups | Foods | Nutritional Supplements |
|---|---|---|
| Cubs | Chicken | Calcium tablets |
| | Chicken rack | |
| | Milk powder | |
| | Egg yolk | |
| Adult and Subadult | Beef | Calcium carbonate |
| | Chicken breast | Vitamin D3 tablets |
| | One egg | Gold theragran, (three times a week) |
| | Live chicken (every Thursday) | |

## Supplemental Table S3. Gut microbiome composition of South China tigers at the phylum level.

| Phylum | M5(%) | M6(%) | M7(%) | M8(%) | M9(%) | M10(%) | M11(%) | M12(%) | Subadult(%). | Adult(%) |
|---|---|---|---|---|---|---|---|---|---|---|
| *Acidobacteriota* | 0.11±0.06$^{a}$ | 0±0 $^{a}$ | 0±0 $^{a}$ | 0±0 $^{a}$ | 0.02±0.02$^{a}$ | 0±0 $^{a}$ | 0±0 $^{a}$ | 0±0 $^{a}$ | 0.12±0.12 $^{a}$ | 0.18±0.14 $^{a}$ |
| *Actinobacteriota* | 3.77±2.20 $^{a}$ | 1.38±0.49 $^{a}$ | 3.52±2.70 $^{a}$ | 6.86±5.17 $^{a}$ | 4.71±5.44$^{a}$ | 3.2±1.42 $^{a}$ | 2.02±1.09$^{a}$ | 4.36±4.01$^{a}$ | 4.38±2.07$^{a}$ | 4.16±1.04 $^{a}$ |
| *Bacteroidota* | 24.41±9.35 $^{a}$ | 8.91±11.09 $^{a}$ | 22.88±18.52$^{a}$ | 23.64±14.47 $^{a}$ | 13.27±8.17$^{a}$ | 6.75±8.32 $^{a}$ | 13.55±9.14 $^{a}$ | 5.8±1.32 $^{a}$ | 14.35±7.22 $^{a}$ | 18.38±7.50 $^{a}$ |
| *Caldisericota* | 0.05±0.03 $^{b}$ | 0±0 $^{a}$ | 0±0 $^{a}$ | 0±0 $^{a}$ | 0±0 $^{a}$ | 0±0 $^{a}$ | 0±0 $^{a}$ | 0±0 $^{a}$ | 0.01±0.01 $^{a}$ | 0.01±0.01 $^{a}$ |
| *Campylobacterota* | 1.55±1.55 $^{a}$ | 12.67±16.52 $^{a}$ | 6.56±6.35$^{a}$ | 3.38±5.09 $^{a}$ | 2.07±2.03$^{a}$ | 0.68±0.82$^{a}$ | 4.97±7.87 $^{a}$ | 0.39±0.35$^{a}$ | 0.57±0.36 $^{a}$ | 0.33±0.18 $^{a}$ |
| *Chloroflexi* | 0.05±0.03 $^{a}$ | 0±0 $^{a}$ | 0.02±0.03 $^{a}$ | 0±0 $^{a}$ | 0.08±0.06$^{a}$ | 0±0.00 $^{a}$ | 0±0.00$^{a}$ | 0±0.00$^{a}$ | 0.13±0.21 $^{a}$ | 0.16±0.15 $^{a}$ |
| *Cyanobacteria* | 0.01±0.01 $^{ab}$ | 0.01±0.00 $^{ab}$ | 0.01±0.01$^{ab}$ | 0.04±0.04$^{ab}$ | 0±0.00$^{ab}$ | 1.3±1.10 $^{c}$ | 0.91±0.59$^{bc}$ | 0.46±0.17$^{abc}$ | 0.01±0.03 $^{a}$ | 0.04±0.03 $^{a}$ |
| *Desulfobacterota* | 0.34±0.22 $^{bc}$ | 0.02±0.03 $^{a}$ | 0.02±0.01 $^{a}$ | 0.01±0.00 $^{a}$ | 0.21±0.14 $^{abc}$ | 0.02±0.01 $^{a}$ | 0.01±0.01 $^{a}$ | 0.01±0.01$^{a}$ | 0.11±0.12 $^{ab}$ | 0.36±0.13 $^{c}$ |
| *Firmicutes* | 27.24±15.13 $^{a}$ | 35.91±26.96 $^{a}$ | 45.74±28.39$^{a}$ | 34.5±20.05$^{a}$ | 33.94±13.99 $^{a}$ | 53.87±18.42 $^{a}$ | 43.86±7.83 $^{a}$ | 55.45±12.56$^{a}$ | 56.82±20.15 $^{a}$ | 45.55±13.43$^{a}$ |
| *Fusobacteriota* | 33.99±11.26$^{a}$ | 36.27±14.02 $^{a}$ | 10.99±7.53 $^{a}$ | 24.41±5.08$^{a}$ | 26.41±20.37 $^{a}$ | 26.92±9.74 $^{a}$ | 23.09±15.84 $^{a}$ | 27.46±14.83 $^{a}$ | 17.25±12.78$^{a}$ | 19.86±13.58 $^{a}$ |
| *Nitrospirota* | 0.1±0.12 $^{a}$ | 0±0 $^{a}$ | 0±0.00 $^{a}$ | 0±0 $^{a}$ | 0±0.00$^{a}$ | 0±0 $^{a}$ | 0±0 $^{a}$ | 0±0 $^{a}$ | 0.06±0.07 $^{a}$ | 0.01±0.01 $^{a}$ |
| *Proteobacteria* | 7.69±2.48$^{ab}$ | 4.32±3.51 $^{a}$ | 9.79±3.99 $^{ab}$ | 6.65±7.53$^{ab}$ | 17.42±7.74$^{b}$ | 6.67±2.12 $^{ab}$ | 9.88±6.39 $^{ab}$ | 5.12±0.83 $^{a}$ | 5.01±2.21 $^{a}$ | 8.22±4.38 $^{ab}$ |
| *Synergistota* | 0.01±0.00 $^{a}$ | 0±0.00 $^{a}$ | 0±0 $^{a}$ | 0±0 $^{a}$ | 0.2±0.15 $^{b}$ | 0±0.00 $^{a}$ | 0±0 $^{a}$ | 0±0 $^{a}$ | 0±0.01$^{a}$ | 0.02±0.01 $^{a}$ |
| *unidentified_Bacteria* | 0.51±0.32 $^{a}$ | 0.5±0.39 $^{a}$ | 0.45±0.27$^{a}$ | 0.49±0.06$^{a}$ | 1.55±0.85$^{a}$ | 0.58±0.1 $^{a}$ | 1.7±0.76 $^{a}$ | 0.94±1.07 $^{a}$ | 0.97±0.86 $^{a}$ | 1.99±0.50 $^{a}$ |
| *Verrucomicrobiota* | 0.01±0.00 $^{a}$ | 0±0.00 $^{a}$ | 0±0 $^{a}$ | 0±0.00$^{a}$ | 0±0.00$^{a}$ | 0.01±0.01$^{a}$ | 0±0 $^{a}$ | 0±0.00$^{a}$ | 0.03±0.04 a | 0.18±0.31 $^{a}$ |
| Other | 0.16±0.08 $^{ab}$ | 0±0.00 $^{a}$ | 0±0.00$^{a}$ | 0.01±0.00 $^{a}$ | 0.12±0.09$^{a}$ | 0±0 $^{a}$ | 0±0 $^{a}$ | 0±0 $^{a}$ | 0.17±0.28 $^{a}$ | 0.57±0.28 $^{b}$ |

Statistical significance between different groups was indicated by a different letter.

**Supplemental Table S4. Gut microbiome composition in different tiger subspecies at the phylum level.**

| Phylum | SC (%) | Bg (%) | Am (%) |
|---|---|---|---|
| *Acidobacteriota* | 0.12±0.13 [a] | 0.08±0.023 [a] | 0.12±0.26 [a] |
| *Actinobacteriota* | 2.62±2.10 [a] | 21.97±20.89 [a] | 3.49±1.31 [a] |
| *Bacteroidota* | 19.8±8.90 [a] | 7.4±4.08 [b] | 14±3.44 [ab] |
| *Campylobacterota* | 0.28±0.16 [a] | 5.04±10.87 [a] | 0.83±1.05 [a] |
| *Desulfobacterota* | 0.18±0.20 [a] | 0.28±0.19[a] | 0.1±0.19 [a] |
| *Firmicutes* | 47.92±13.55 [a] | 50.55±27.49 [a] | 59.36±4.24 [a] |
| *Fusobacteriota* | 19.22±12.68 [a] | 8.2±5.19 [a] | 16.99±7.87 [a] |
| *Halobacterota* | 0±0.00 [a] | 0.06±0.04 [a] | 0.09±0.20 [a] |
| *Nitrospirota* | 0.31±0.68[a] | 0.08±0.05 [a] | 0.15±0.29 [a] |
| *Proteobacteria* | 7.77±5.13 [a] | 4.73±2.83 [a] | 2.86±2.06 [a] |
| *Verrucomicrobiota* | 0.33±0.14 [a] | 0.01±0.01[a] | 0.35±0.42 [a] |
| Other | 1.45±0.46 [a] | 1.61±0.80 [a] | 1.66±1.63 [a] |

Statistical significance between different groups was indicated by a different letter.

**Supplemental Table S5. Gut microbiome composition of South China tigers at the genus level.**

| Genus | M5(%) | M6(%) | M7(%) | M8(%) | M9(%) | M10(%) | M11(%) | M12(%) | Subadult(%) | Adult(%) |
|---|---|---|---|---|---|---|---|---|---|---|
| *Anaerobiospirillum* | 0±0 $^{a}$ | 2.31±3.55 $^{a}$ | 0.22±0.08 $^{a}$ | 0.59±0.83 $^{a}$ | 0±0.00 $^{a}$ | 0.05±0.00 $^{a}$ | 0.27±0.41 $^{a}$ | 0.02±0.02 $^{a}$ | 0±0 $^{a}$ | 0±0 $^{a}$ |
| *Bacteroides* | 22.78±10.03 $^{a}$ | 6.1±6.67 $^{a}$ | 22.09±18.58 $^{a}$ | 22.22±14.40$^{a}$ | 10.91±7.75$^{a}$ | 6.2±7.92$^{a}$ | 13.42±9.14 $^{a}$ | 4.77±0.47 $^{a}$ | 12.03±7.16 $^{a}$ | 15.32±7.59 $^{a}$ |
| *Blautia* | 2.43±2.75 $^{a}$ | 1.61±0.63 $^{a}$ | 2.11±2.50 $^{a}$ | 1.78±1.74 $^{a}$ | 1.12±0.80 $^{a}$ | 2.19±0.34 $^{a}$ | 4.71±1.36 $^{a}$ | 4.14±4.63 $^{a}$ | 3.08±2.49 $^{a}$ | 1.53±0.76 $^{a}$ |
| *Campylobacter* | 1.53±1.53 $^{a}$ | 12.65±16.50 $^{a}$ | 5.96±5.61 $^{a}$ | 3.13±4.95 $^{a}$ | 1.99±1.92 $^{a}$ | 0.56±0.65 $^{a}$ | 4.92±7.80 $^{a}$ | 0.36±0.33 $^{a}$ | 0.52±0.35 $^{a}$ | 0.26±0.16 $^{a}$ |
| *Clostridium_sensu_stricto_1* | 6.66±6.58 $^{a}$ | 19.36±16.85 $^{a}$ | 2.37±0.73 $^{a}$ | 4.52±5.20 $^{a}$ | 12.3±6.74 $^{a}$ | 13.12±3.68$^{a}$ | 14.72±8.15 $^{a}$ | 20.34±7.94 $^{a}$ | 22.53±10.78$^{a}$ | 17.65±8.00 $^{a}$ |
| *Clostridium_sensu_stricto_4* | 0.02±0.01 $^{a}$ | 0.16±0.21 $^{ab}$ | 0±0.00$^{a}$ | 0.01±0.01 $^{a}$ | 0±0.00$^{a}$ | 0.58±0.34 $^{ab}$ | 0.35±0.19 $^{ab}$ | 0.34±0.13 $^{ab}$ | 0.35±0.21 $^{ab}$ | 0.89±0.76 $^{b}$ |
| *Collinsella* | 3.48±2.19 $^{a}$ | 1.12±0.44 $^{a}$ | 3.09±2.62 $^{a}$ | 6.47±5.08 $^{a}$ | 4.14±5.64 $^{a}$ | 2.52±1.44 $^{a}$ | 1.45±0.84 $^{a}$ | 3.39±3.53 $^{a}$ | 3.18±2.36 $^{a}$ | 1.78±1.47 $^{a}$ |
| *Enterococcus* | 0.04±0.04 $^{a}$ | 0.15±0.11 $^{a}$ | 0.09±0.11 $^{a}$ | 0.41±0.54 $^{a}$ | 0.15±0.11 $^{a}$ | 0.29±0.39 $^{a}$ | 0.39±0.14 $^{a}$ | 0.38±0.45 $^{a}$ | 0.07±0.06 $^{a}$ | 0.1±0.19 $^{a}$ |
| *Escherichia.Shigella* | 2.93±1.58 $^{a}$ | 0.25±0.10 $^{a}$ | 0.22±0.07 $^{a}$ | 0.25±0.20 $^{a}$ | 13.27±9.16 $^{b}$ | 0.67±0.39 $^{a}$ | 0.41±0.28 $^{a}$ | 1.38±0.58 $^{a}$ | 2.71±1.92 $^{a}$ | 3.89±3.01 $^{a}$ |
| *Fusobacterium* | 33.98±11.26$^{a}$ | 36.26±14.02 $^{a}$ | 10.96±7.53 $^{a}$ | 24.37±5.08 $^{a}$ | 26.41±20.37$^{a}$ | 23.92±11.56$^{a}$ | 21.8±14.37 $^{a}$ | 27.46±14.83 $^{a}$ | 17.25±12.78$^{a}$ | 19.86±13.58$^{a}$ |
| *Lachnoclostridium* | 0.64±0.31 $^{a}$ | 0.6±0.07 $^{a}$ | 2.62±2.42 $^{a}$ | 2.7±2.65 $^{a}$ | 2.13±1.54 $^{a}$ | 2.26±0.93 $^{a}$ | 5.04±0.83 $^{a}$ | 1.92±1.49 $^{a}$ | 2.65±2.33 $^{a}$ | 2.32±1.01 $^{a}$ |
| *Paeniclostridium* | 1.34±0.63 $^{a}$ | 8.72±12.25 $^{a}$ | 0.11±0.03 $^{a}$ | 0.73±1.01 $^{a}$ | 2.7±2.05 $^{a}$ | 3.17±1.46 $^{a}$ | 4.96±5.04 $^{a}$ | 1.57±0.69 $^{a}$ | 9.47±5.96 $^{a}$ | 9.06±4.34 $^{a}$ |
| *Peptoclostridium* | 3.46±1.70 $^{a}$ | 1.26±1.10 $^{a}$ | 0.41±0.22 $^{a}$ | 0.39±0.32 $^{a}$ | 3.73±4.59 $^{a}$ | 2.32±0.93 $^{a}$ | 2.06±0.62 $^{a}$ | 1.83±0.93 $^{a}$ | 3.69±2.14 $^{a}$ | 2.27±1.53 $^{a}$ |
| *Peptoniphilus* | 0.05±0.02 $^{ab}$ | 0.01±0.00 $^{ab}$ | 0.03±0.04 $^{ab}$ | 0±0.00$^{ab}$ | 0±0.00$^{ab}$ | 0.25±0.05 $^{ab}$ | 1.61±2.09 $^{b}$ | 0.54±0.51 $^{ab}$ | 0.1±0.08 $^{a}$ | 0.14±0.23 $^{ab}$ |
| *Solobacterium* | 0.01±0.01 $^{ab}$ | 0.2±0.34 $^{ab}$ | 30.55±33.19 $^{b}$ | 17.99±15.79$^{ab}$ | 5.22±9.02 $^{ab}$ | 21.58±16.79$^{ab}$ | 0.9±0.27 $^{ab}$ | 15.75±20.94$^{ab}$ | 0.59±0.79 $^{a}$ | 0.18±0.22 $^{ab}$ |
| *Sutterella* | 1.94±2.06 $^{a}$ | 0.49±0.54 $^{a}$ | 4.77±5.10 $^{a}$ | 4.74±6.79 $^{a}$ | 2.24±2.86 $^{a}$ | 1.87±2.01 $^{a}$ | 5.25±5.75 $^{a}$ | 1.18±0.30 $^{a}$ | 0.54±0.29 $^{a}$ | 1.24±1.57 $^{a}$ |
| *X.Ruminococcus._gnavus_group* | 2.92±3.84 $^{a}$ | 0.85±0.71 $^{a}$ | 1.69±1.13 $^{a}$ | 1.15±0.11 $^{a}$ | 1.24±0.38 $^{a}$ | 2.15±0.56 $^{a}$ | 3.44±1.16 $^{a}$ | 3.27±2.94 $^{a}$ | 7.48±11.81 $^{a}$ | 1.79±0.56 $^{a}$ |
| Other | 15.79±3.70 $^{ab}$ | 7.91±4.05 $^{a}$ | 12.72±6.46 $^{ab}$ | 8.54±3.74 $^{a}$ | 12.39±4.42 $^{ab}$ | 16.32±4.44 $^{ab}$ | 14.32±1.79 $^{ab}$ | 11.36±2.22 $^{a}$ | 13.66±4.66 $^{a}$ | 21.7±2.26 $^{b}$ |

Statistical significance between different groups was indicated by a different letter.

**Supplemental Table S6. Gut microbiome composition in different tiger subspecies at the genus level.**

| Genus | SC (%) | Bg (%) | Am (%) |
|---|---|---|---|
| *Bacteroides* | 15.45±7.86 [a] | 6.18±4.06 [a] | 9.62±4.42 [a] |
| *Blautia* | 1.65±0.80 [a] | 2.04±1.13 [a] | 1.96±0.96 [a] |
| *Campylobacter* | 0.24±0.17 [a] | 4.97±10.73 [a] | 0.8±1.06 [a] |
| *Clostridium_sensu_stricto_1* | 17.76±8.32 [a] | 19.58±21.24 [a] | 24.7±6.00 [a] |
| *Collinsella* | 1.8±1.52 [a] | 21.35±20.76 [a] | 3.01±1.09 [a] |
| *Escherichia.Shigella* | 3.8±2.94 [a] | 2.35±2.61 [a] | 0.92±0.60 [a] |
| *Fusobacterium* | 19.22±12.68 [a] | 8.2±5.19 [a] | 16.99±7.87 [a] |
| *Lachnoclostridium* | 1.59±1.36 [a] | 1.12±0.69 [a] | 2.32±1.56 [a] |
| *Paeniclostridium* | 8.85±4.36 [a] | 7±8.63 [a] | 11.8±1.67 [a] |
| *Peptoclostridium* | 2.24±1.56 [a] | 5.48±6.20 [a] | 3.09±1.17 [a] |
| *Romboutsia* | 1.32±0.80 [a] | 0.15±0.21 [b] | 1.19±0.52 [a] |
| *Solobacterium* | 0.22±0.26 [a] | 4.04±8.95 [a] | 0.74±0.35 [a] |
| *X.Ruminococcus._gnavus_group* | 1.75±0.56 [a] | 4.9±5.18 [a] | 1.39±0.70 [a] |
| Other | 24.11±4.59 [a] | 12.63±4.50 [a] | 21.45±11.23 [a] |

Statistical significance between different groups was indicated by a different letter.

**Supplemental Table S7. Core OTUs shared by all South China tigers.**

| Id | Phylum | Genus | M5(%) | M6(%) | M7(%) | M8(%) | M9(%) | M10(%) | M11(%) | M12(%) | Subadult (%) | Adult (%) |
|---|---|---|---|---|---|---|---|---|---|---|---|---|
| Out_1 | *Firmicutes* | *Clostridium_sensu_stricto_1* | 5.72 | 18.81 | 2.34 | 4.36 | 11.21 | 10.52 | 11.24 | 17.14 | 20.14 | 13.54 |
| Out_2 | *Fusobacteriota* | *Fusobacterium* | 15.62 | 27.74 | 3.98 | 11.12 | 12.06 | 19.52 | 17.59 | 24.53 | 13.95 | 17.32 |
| Out_4 | *Campylobacterota* | *Campylobacter* | 1.52 | 12.62 | 5.95 | 3.13 | 1.99 | 0.56 | 4.92 | 0.36 | 0.51 | 0.24 |
| Out_7 | *Fusobacteriota* | *Fusobacterium* | 11.58 | 6.22 | 4.76 | 10.23 | 6.87 | 3.32 | 3.88 | 2.08 | 1.78 | 1.77 |
| Out_8 | *Bacteroidota* | *Bacteroides* | 14.91 | 5.26 | 12.28 | 8.62 | 0.92 | 2.00 | 5.63 | 1.66 | 2.11 | 2.02 |
| Out_9 | *Firmicutes* | *[Ruminococcus]_gnavus_group* | 2.88 | 0.84 | 1.66 | 1.15 | 1.23 | 2.14 | 3.43 | 3.26 | 7.43 | 1.76 |
| Out_10 | *Proteobacteria* | *Escherichia-Shigella* | 2.92 | 0.25 | 0.22 | 0.25 | 13.26 | 0.67 | 0.41 | 1.38 | 2.69 | 3.80 |
| Out_11 | *Firmicutes* | *Lachnoclostridium* | 0.38 | 0.40 | 2.42 | 2.07 | 1.90 | 1.77 | 4.76 | 1.69 | 2.27 | 1.47 |
| Out_12 | *Firmicutes* | *Blautia* | 2.13 | 1.36 | 1.59 | 0.67 | 0.55 | 1.36 | 3.75 | 3.24 | 2.83 | 1.07 |
| Out_13 | *Firmicutes* | *Peptoclostridium* | 3.44 | 1.26 | 0.41 | 0.39 | 3.72 | 2.31 | 2.06 | 1.83 | 3.66 | 2.24 |
| Out_15 | *Actinobacteriota* | *Collinsella* | 0.63 | 0.41 | 2.23 | 4.64 | 1.45 | 1.97 | 0.62 | 2.38 | 2.56 | 1.10 |
| Out_16 | *Proteobacteria* | *Sutterella* | 1.94 | 0.49 | 4.76 | 4.73 | 2.24 | 1.86 | 5.21 | 1.15 | 0.53 | 1.21 |

**Supplemental Table S8. Core OTUs shared by all tiger subspecies.**

| Id | Phylum | Genus | SC (%) | Bg (%) | Am (%) |
|---|---|---|---|---|---|
| Out_1 | *Firmicutes* | *Clostridium_sensu_stricto_1* | 13.63 | 18.18 | 20.77 |
| Out_2 | *Fusobacteriota* | *Fusobacterium* | 16.86 | 4.17 | 14.84 |
| Out_3 | *Firmicutes* | *Paeniclostridium* | 8.85 | 7.00 | 11.80 |
| Out_5 | *Firmicutes* | *Peptoclostridium* | 2.24 | 5.48 | 3.09 |
| Out_6 | *Firmicutes* | *Lachnoclostridium* | 1.47 | 1.08 | 2.29 |
| Out_7 | *Firmicutes* | *[Ruminococcus]_gnavus_group* | 1.74 | 4.87 | 1.38 |
| Out_9 | *Firmicutes* | *Blautia* | 1.07 | 1.58 | 1.41 |
| Out_10 | *Fusobacteriota* | *Fusobacterium* | 1.76 | 1.94 | 1.54 |
| Out_13 | *Actinobacteriota* | *Collinsella* | 1.11 | 16.01 | 1.79 |
| Out_14 | *Fusobacteriota* | *Fusobacterium* | 0.60 | 2.09 | 0.62 |
| Out_16 | *Firmicutes* | *Clostridium_sensu_stricto_1* | 3.24 | 1.13 | 3.39 |
| Out_22 | *Bacteroidota* | *Bacteroides* | 0.57 | 0.60 | 0.91 |
| Out_1395 | NA | NA | 0.32 | 1.07 | 0.41 |

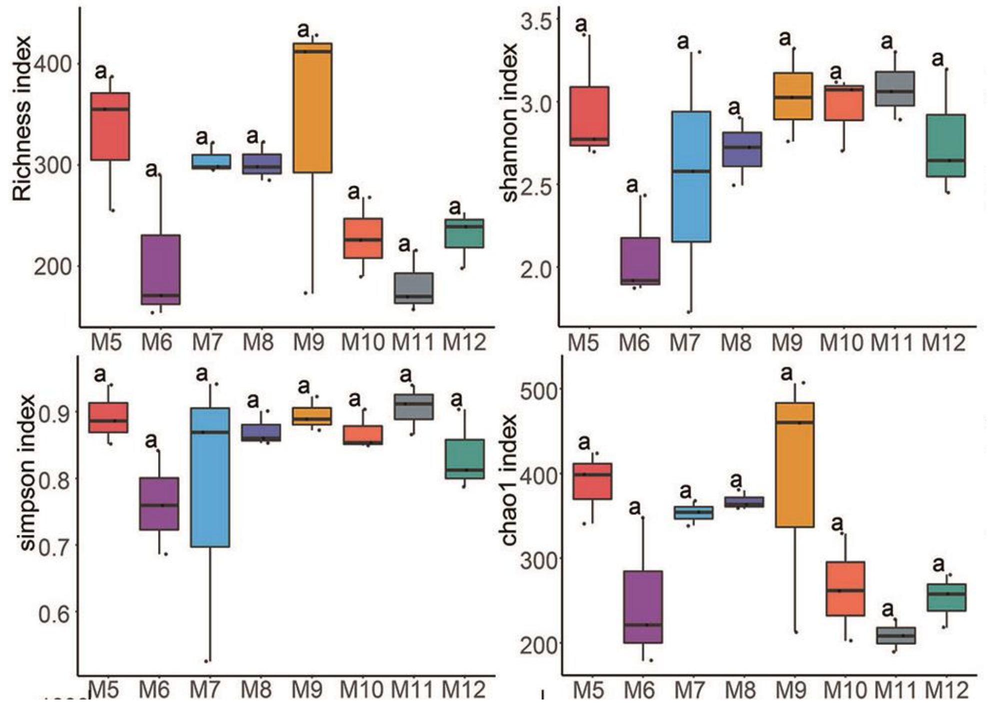


**Supplemental Figure S1 Alpha diversity of gut microbiota in Juvenile South China tigers.** The evenness of the bacteria community was evaluated by the Shannon and Simpson index, and the richness of the bacteria community was evaluated by the Richness and Chao1 index. Box plots show high, low, and median values, with the lower and upper edges of each box denoting the first and third quartiles, respectively. The *x*-axis represents the information of samples, and M5-M12 indicated the group of the 5-12 months old tigers. Statistical significance between different groups was indicated by a different letter ($P < 0.05$, ANOVA)

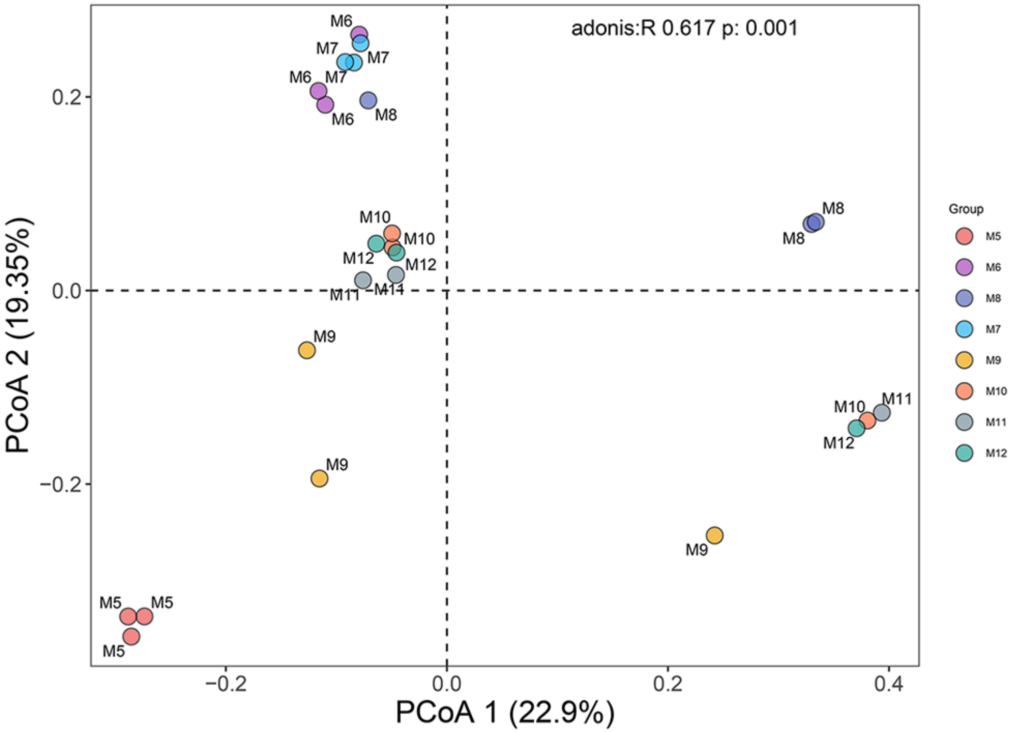


**Supplemental Figure S2 Beta diversity of gut microbiota in Juvenile South China tigers.** Beta diversity is shown by Principal coordinate analyses (PCoA) based on Unweight Unifrac distance at the operational taxonomic unit (OTU) level. The variation explained by the plotted principal coordinates is indicated in the axis label. *p*-values of Adnois tests after adjusting by FDR are noted at the top of each PCoA plot.

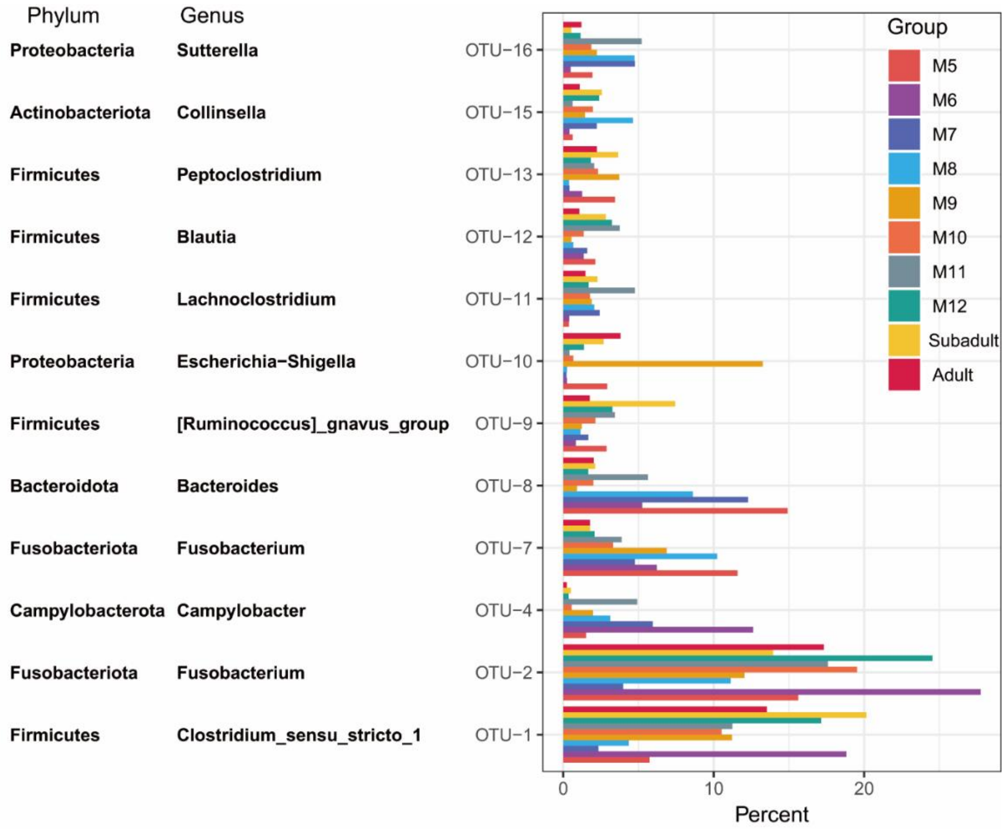


**Supplemental Figure S3 Core OTUs shared by all South China tigers.**

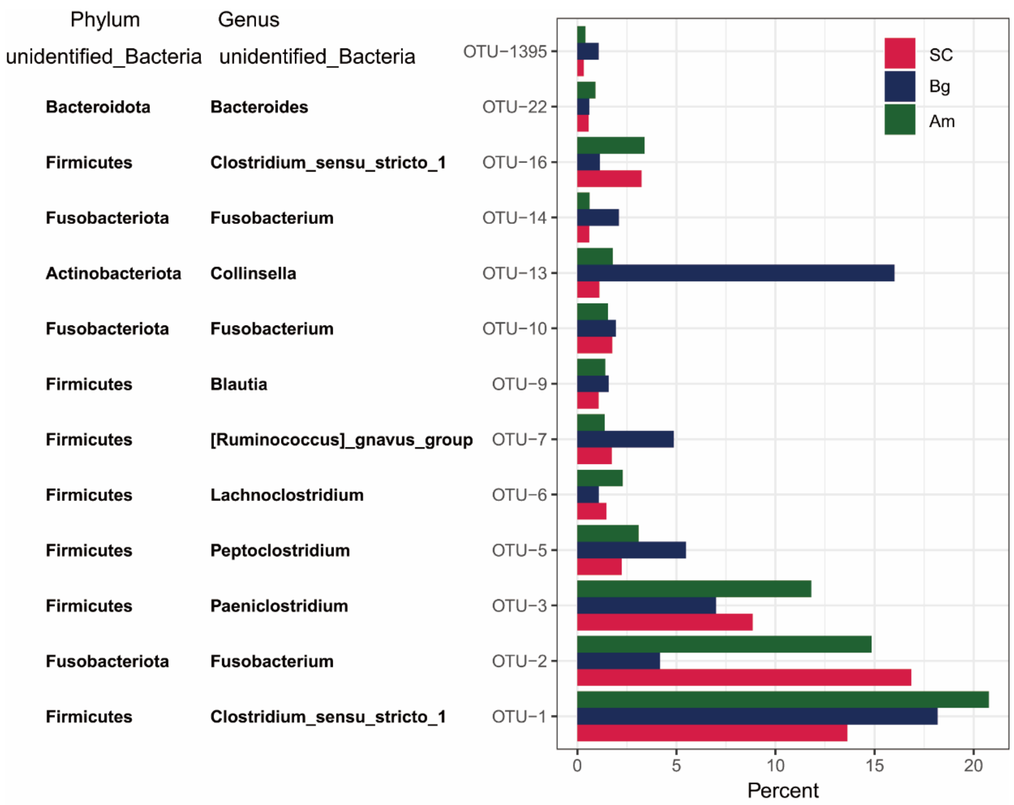


**Supplemental Figure S4 Core OTUs shared by all tiger subspecies.**
